# Exploring the link between microseism and sea ice in Antarctica by using machine learning

**DOI:** 10.1038/s41598-019-49586-z

**Published:** 2019-09-10

**Authors:** Andrea Cannata, Flavio Cannavò, Salvatore Moschella, Stefano Gresta, Laura Spina

**Affiliations:** 1 Università degli Studi di Catania, Dipartimento di Scienze Biologiche, Geologiche e Ambientali - Sezione di Scienze della Terra, Corso Italia 57, I-95129 Catania, Italy; 2 Istituto Nazionale di Geofisica e Vulcanologia, Osservatorio Etneo – Sezione di Catania, Piazza Roma 2, 95123 Catania, Italy; 3 Istituto Nazionale di Geofisica e Vulcanologia, Sezione di Roma 1, Via di Vigna Murata, 605, 00143 Roma, RM Italy

**Keywords:** Geophysics, Seismology

## Abstract

The most continuous and ubiquitous seismic signal on Earth is the microseism, closely related to ocean wave energy coupling with the solid Earth. A peculiar feature of microseism recorded in Antarctica is the link with the sea ice, making the temporal pattern of microseism amplitudes different with respect to the microseism recorded in low-middle latitude regions. Indeed, during austral winters, in Antarctica the oceanic waves cannot efficiently excite seismic energy because of the sea ice in the Southern Ocean. Here, we quantitatively investigate the relationship between microseism, recorded along the Antarctic coasts, and sea ice concentration. In particular, we show a decrease in sea ice sensitivity of microseism, due to the increasing distance from the station recording the seismic signal. The influence seems to strongly reduce for distances above 1,000 km. Finally, we present an algorithm, based on machine learning techniques, allowing to spatially and temporally reconstruct the sea ice distribution around Antarctica based on the microseism amplitudes. This technique will allow reconstructing the sea ice concentration in both Arctic and Antarctica in periods when the satellite images, routinely used for sea ice monitoring, are not available, with wide applications in many fields, first of all climate studies.

## Introduction

Modern seismology is able to obtain plenty of information by the analyses of signals, that until a couple of decades ago were considered to be noise, such as microseism. This, considered as the most continuous and ubiquitous seismic signal on Earth, is generated by ocean wave energy coupling with the Earth’s ground^1–3^ and is generally classified as primary and secondary^4^. Primary microseism has a spectral content equal to the ocean wave frequency (period between 13 and 20 s) and its source is associated with the energy transfer of ocean waves breaking/shoaling against the shoreline^2^. Secondary microseism, with most energy between 5 and 10 s (roughly twice the frequency of ocean waves)^5^, is generally characterised by higher amplitude than primary microseism. According to the most accredited theories, secondary microseism is generated by interactions between waves of the same frequency travelling in opposite directions^1,6^. Finally, there is a short period secondary microseism, characterized by period shorter than 5 s and sources generally linked to local sea state and wave activity, and influenced by local winds^7,8^.

Microseism studies have today broad applications, such as the reconstruction of crust and upper mantle by noise tomography^9,10^ and the detection of seismic velocity variations in both volcanic and tectonic area^11,12^. Furthermore, because of the microseism source nature, such a signal has been used to make inferences on climate changes^13–15^.

Microseism amplitudes at temperate latitudes in both northern and southern hemispheres show strong annual periodicity with maxima during the winter seasons, when the oceans are stormier, and minima during summers^14^. However, such a pattern is different in Antarctica where during the winter, because of the sea ice, the oceanic waves cannot efficiently excite seismic energy^14–18^. Although the link between microseism and ocean wave parameters has been quantitatively explored^6,19,20^, the relationship between sea ice concentration and microseism recorded in Antarctica has only been qualitatively treated. An exception is the investigation performed by^18^, who quantitatively studied such a relationship only in the Antarctic Peninsula. In addition, to date no technique has been found to try to infer the sea ice distribution in the Southern Ocean, based on microseism recordings.

Such investigations, that have to face the processing of decade-long seismic signals as well as the lack of precise models dealing with the multi-dimensional complexity inherent in the data of such extreme environments, are really challenging. The modern data mining and machine learning techniques can help extract implicit useful information and knowledge from a large quantity of data, allowing to unravel hidden relationships between parameters. Recent applications of data mining and machine learning techniques to geosciences have regarded many topics such as seismic signal classification^21^, remote sensing^22^, volcano monitoring^23^ and earthquake studies^24^.

## Results

### Microseism modulation by sea ice

**Figure 1:**
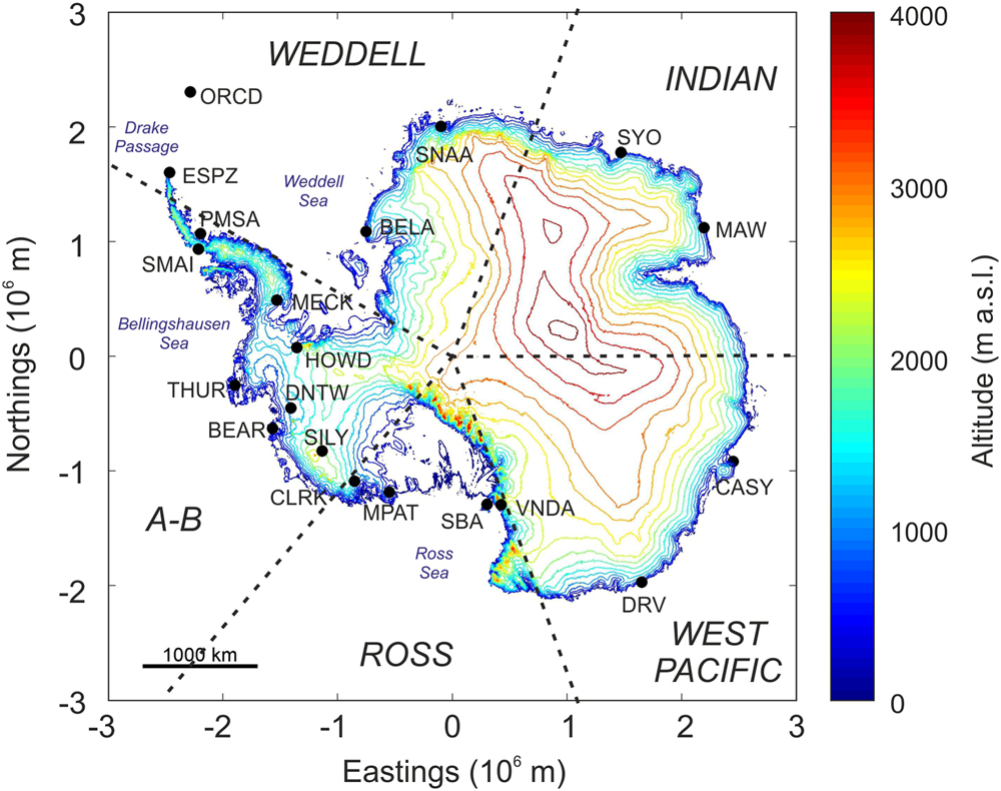
Antarctica map. Digital elevation model of Antarctica (data derived from CryoSat-2 altimetry^62^); plotted by the Antarctic Mapping Tools^60^, showing the locations of the seismic stations used in this study. The dashed black lines divide Antarctica into 5 sectors named Weddell, Indian, West Pacific, Ross and A-B (acronym of Amundsen-Bellinghausen)^63^.

All the available seismic data, recorded by the vertical component of 20 stations from 1993 to 2017, were used. These stations, chosen because located close to the Antarctica grounding lines (maximum distance equal to ~260 km for SILY) and then to those microseism sources, mainly related to the energy transfer of ocean waves breaking/shoaling against the shoreline (primary microseism)^2^ or to local nearshore wave-wave interaction (short period secondary microseism)^7^ (Fig. 1), belong to the following seismic networks: Antarctic Seismographic Argentinean Italian Network (BELA, ESPZ, ORCD, SMAI), POLENET (BEAR, CLRK, DNTW, HOWD, MECK, MPAT, SILY, THUR), Global Seismograph Network (CASY, PMSA, SBA, VNDA), GEOSCOPE (DRV), Geoscience Australia (MAW), GEOFON (SNAA), Pacific21 (SYO). The data were acquired at a sampling rate of 20 or 40 Hz by seismic stations equipped with different broadband seismometers (Guralp CMG-3T, Streckeisen STS2, Streckeisen STS1, Nanometrics Trillium 240, Geotech KS-54000). The data were downloaded from the website of Incorporated Research Institutions for Seismology (IRIS; https://ds.iris.edu/SeismiQuery/station.htm). Although the considered time interval ranges from 1993 to 2017, the temporal coverage of the acquired data significantly varies from station to station.

**Figure 2:**
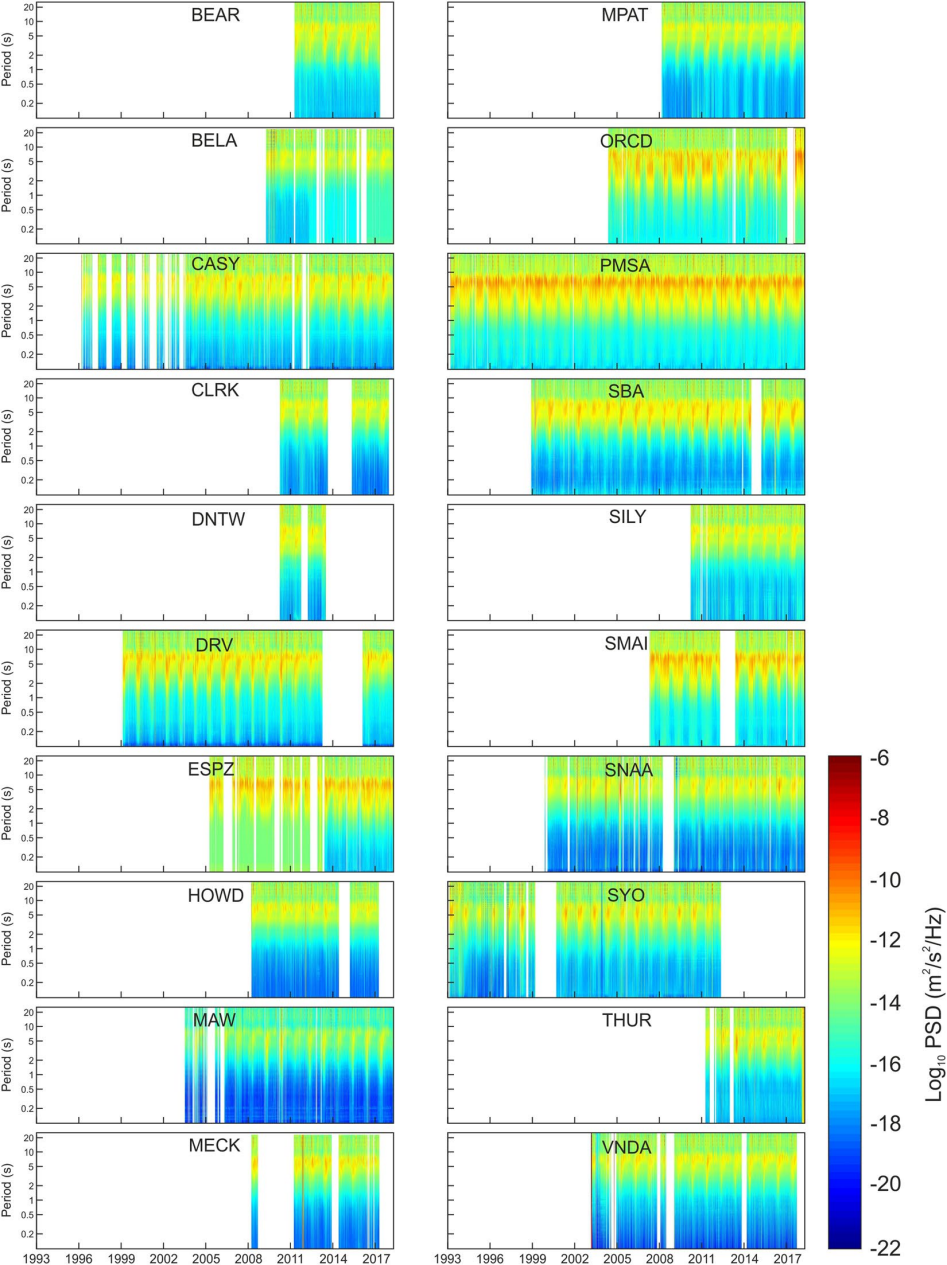
Seismic spectrograms. Spectrograms of the seismic signal recorded by the vertical component of the 20 considered stations.

Once the data were downloaded, they were corrected for the instrument response, and spectrograms (Fig. 2) and RMS amplitude time series (Fig. S1) were computed. As shown in the yearly smoothed and stacked RMS amplitude time series (Fig. S2), microseism exhibits seasonal variability with maxima during February–April (austral fall) and minima during October–December (austral spring-summer). There are slight differences in the seasonal pattern, regarding both the times of maxima and minima (indeed, there are clear lags between the different time series) and the shape of the patterns (Fig. S2a). Such a variability to a first approximation depends on the sector where the stations are located (see Fig. 1 for sector view). For instance, stations falling in the Weddell sector show RMS amplitude maxima for the band 2.5–5.0 s during the period end of January – end of April, while stations in Ross sector exhibit maxima slightly before, mid-January – mid-April (see Fig. 1 for sector explanation). In addition, stations located in the West Pacific sector (and in particular CASY) show a shorter period of minimum RMS amplitudes, with respect to the stations located in the Ross sector. Finally, a certain variability can also be observed among stations belonging to the same sector. The annual patterns of ESPZ and ORCD are evidently different from the patterns shown by the other stations of the Weddell sector.

**Figure 3:**
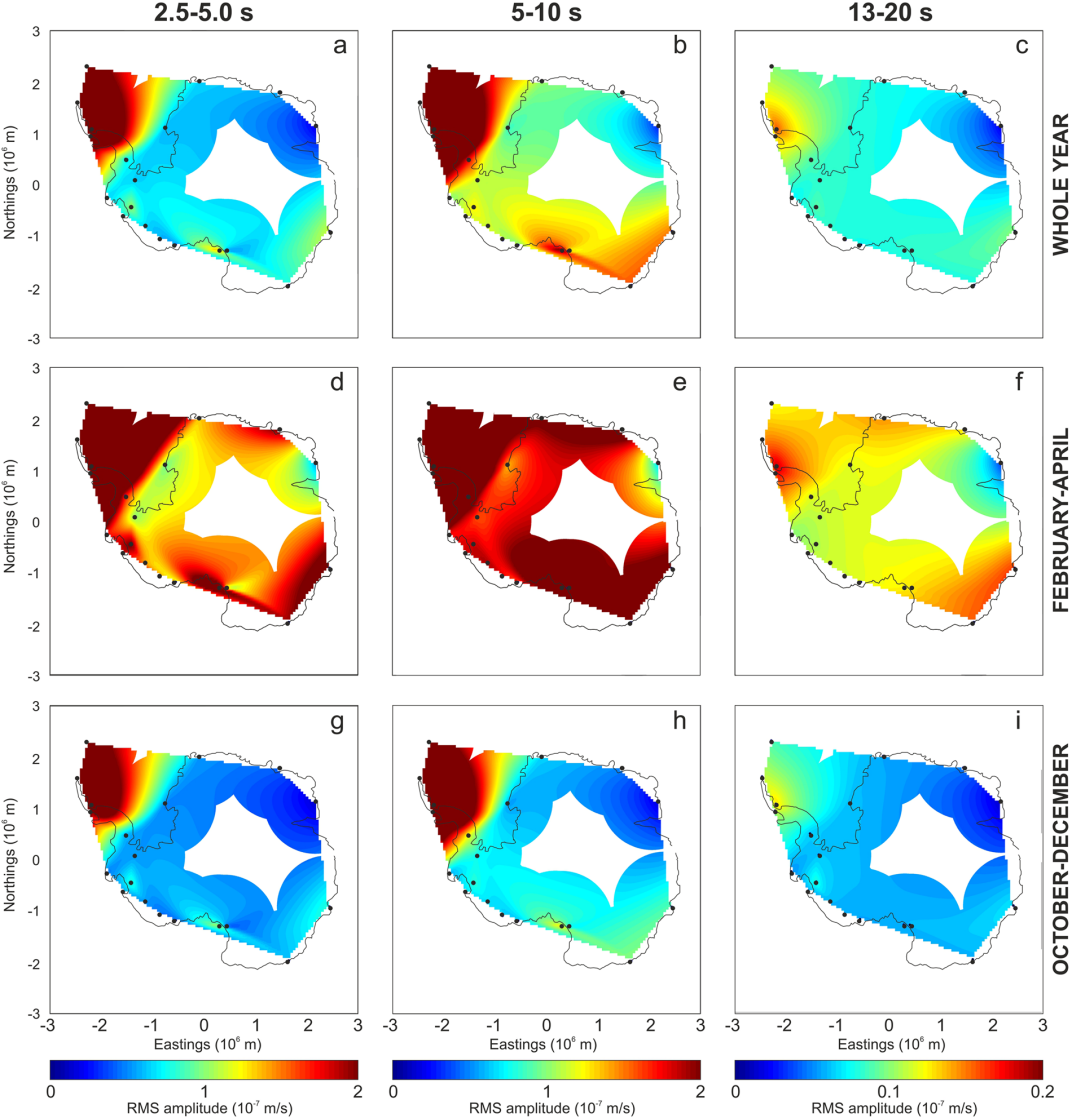
Microseism amplitude maps. Spatial distribution of the median values of RMS amplitude time series in the bands 2.5–5 s (short period secondary microseism; **a**,**d**,**g**), 5–10 s (secondary microseism; **b**,**e**,**h**) and 13–20 s (primary microseism; **c**,**f**,**i**), obtained by applying a triangulation-based natural neighbour interpolation^51^. The plots (**a**–**c**) were obtained by taking into account the whole year, while the plots (**d**–**f**) and (**g**–**i**) are focused on the periods with the strongest (February–April) and weakest (October–December) microseism, respectively. The black dots indicate the locations of the considered stations. It has to be noted that the color scale used for primary microseism 13–20 s (**c**,**f**,**i**) is different from the color scales used for secondary microseism 5–10 s (**b**,**e**,**h**) and short period secondary microseism 2.5–5 s (**a**,**d**,**g**).

The spectrograms show how most microseism energy is comprised in the bands 2.5–5.0 and 5–10 s (short period secondary microseism and secondary microseism, respectively) at all the considered stations (Fig. 2). The different energy content in the distinct period bands is also evident in the maps, showing the spatial distribution of the median value of RMS amplitudes (Fig. 3a–c). Moreover, these maps highlight that the area with maximum microseism amplitude is the West Antarctica, and in particular the Antarctic Peninsula. This feature has also been noted by^17^, who analysed a shorter time period between 2007 and 2012, and interpreted this feature as due to the circumpolar westerlies, making Drake Passage and Bellingshausen Sea very stormy. Strong microseism sources located in this area have also been reported in other papers^25,26^. Moreover, the maps, displaying the spatial distribution of the median value of RMS amplitudes in the three investigated period bands (2.5–5.0, 5–10 and 13–20 s) during February–April (Fig. 3d–f) and October–December (Fig. 3g–i), confirm the strong seasonal modulation of the microseism: higher amplitude during austral fall, and lower amplitude during austral spring-summer.

The microseism RMS amplitudes within the three period bands were compared by Spearman correlation coefficient to the sea ice concentration on the whole Southern Ocean. Information about temporal and spatial variability of sea ice concentration, defined as the percentage of ice cover within each 25 × 25 km^2^ cell of a grid comprising the entire Antarctic polar sea ice cover, are obtained by brightness temperature data^27^. Such data were downloaded as GeoTIFF files, providing the daily sea ice concentration data in a georeferenced format. In particular, the version 2.1 files were used for the period 2000–2016, the version 3.0 files for the period 1993–1999 and 2017. The two versions have no difference in terms of daily sea ice concentration^28^. The sea ice concentration is represented with a scale ranging from 0 to 1000. We divided this value by 10 to get data in percent. It has to be noted that values lower than 150 (15%) are considered statistically irrelevant because of instrumental limits. Such a parameter is affected by larger uncertainty during summer, because of the thinner sea ice^29^.

**Figure 4:**
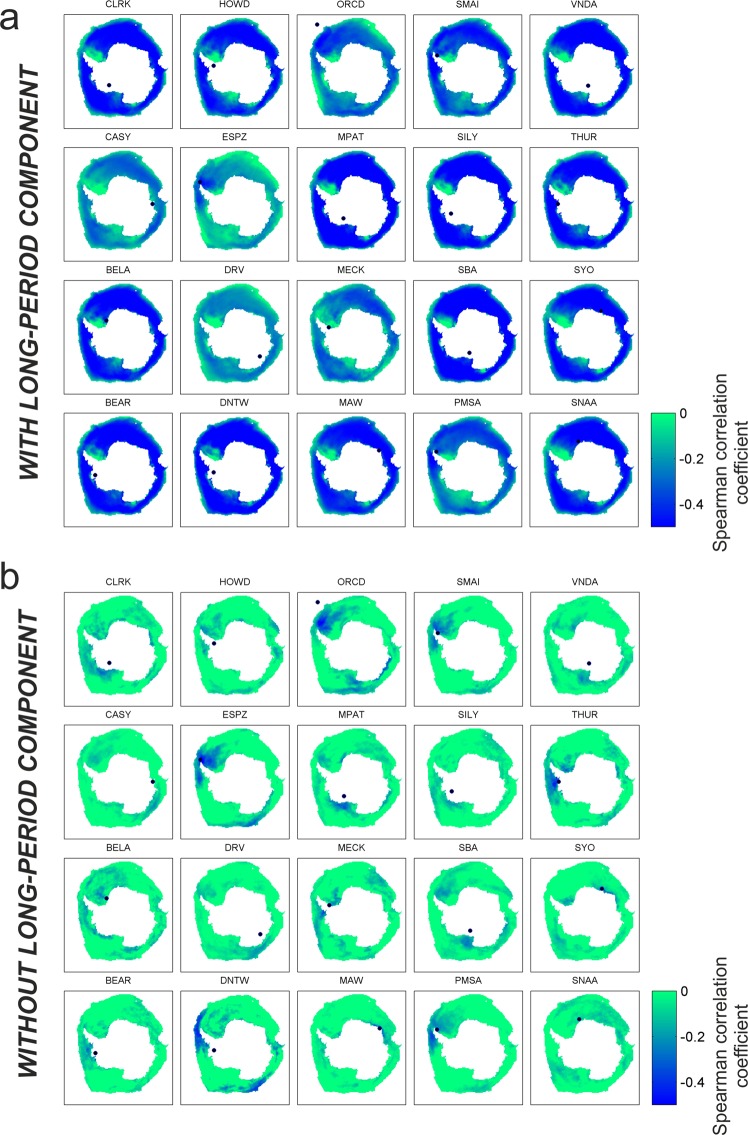
Spearman correlation coefficient maps for short period secondary microseism. Maps of Antarctica showing the space distribution of the Spearman correlation coefficient computed between sea ice concentration time series and RMS amplitudes, calculated per each considered station in the band 2.5–5.0 s. In (**a**) the long-period components of the time series were present, in (**b**) the long-period components were filtered out before performing the correlation analysis. The black dots indicate the locations of the considered stations.

The results of the correlation analysis highlight clear anti-correlation patterns for all the bands (Figs 4a, S3a,c). It is also worth noting that the values of Spearman correlation strongly depend on the considered station and period band. In particular, the period band showing the strongest anti-correlation is 2.5–5.0 s (short period secondary microseism; Fig. 4a). Indeed, the median value calculated on the Spearman correlation minima obtained for all the stations is equal to −0.63 for 2.5–5.0 s band, while it is equal to −0.43 and −0.40 for 5–10 s and 13–20 s (secondary and primary microseism), respectively. Furthermore, the estimated space distribution of the p-values highlights how the anti-correlation obtained for most of the stations is significantly different from zero in wide areas, in some cases coinciding with almost the whole Southern Ocean (Fig. S4a,c,e). This is due to the fact that both the parameters show strong seasonal periodicity.

In addition, to seek a prospective spatial dependence between sea ice concentration and the location of the station recording microseism, the long-period components (among which the seasonal modulation) were filtered out and the correlation analysis was performed again between the filtered time series (Figs 4b, S3b,d and S4b,d,f). The correlation maps show less strong anti-correlations: the median value calculated on the Spearman correlation minima obtained for all the stations is equal to −0.34, −0.28 and −0.29 for 2.5–5.0, 5–10 and 13–20 s, respectively. More importantly, in most cases the Spearman correlation coefficient displays the lowest values in the areas close to the stations where the microseism is recorded. This is evident in many regions, such as the Drake Passage, Bellingshausen Sea and Weddell Sea (i.e. stations ESPZ, HOWD, ORCD, PMSA, SMAI), and the Ross Sea (i.e. stations MPAT, SBA, VNDA) (Figs 1, 4b and S3b,d). According to^18^, in regions with strong anti-correlation we expect that sea ice interferes with the microseism generation.

In a few cases, it is also possible to note low values of Spearman correlation coefficient in areas far away from the stations recording the microseism. This is especially evident for stations located on or nearby the Antarctica Peninsula, such as ORCD, ESPZ and DNTW (see Figs 1 and 4b), whose Spearman correlation maps show anti-correlation both close to the stations (Weddell Sea and/or Bellinghausen Sea) and far away (mostly Western Ross Sea). However, such an apparent link between microseism and very distant sea ice is due to the fact that time series of sea ice concentration in areas far away from each other can have similar patterns (Fig. S5).

**Figure 5:**
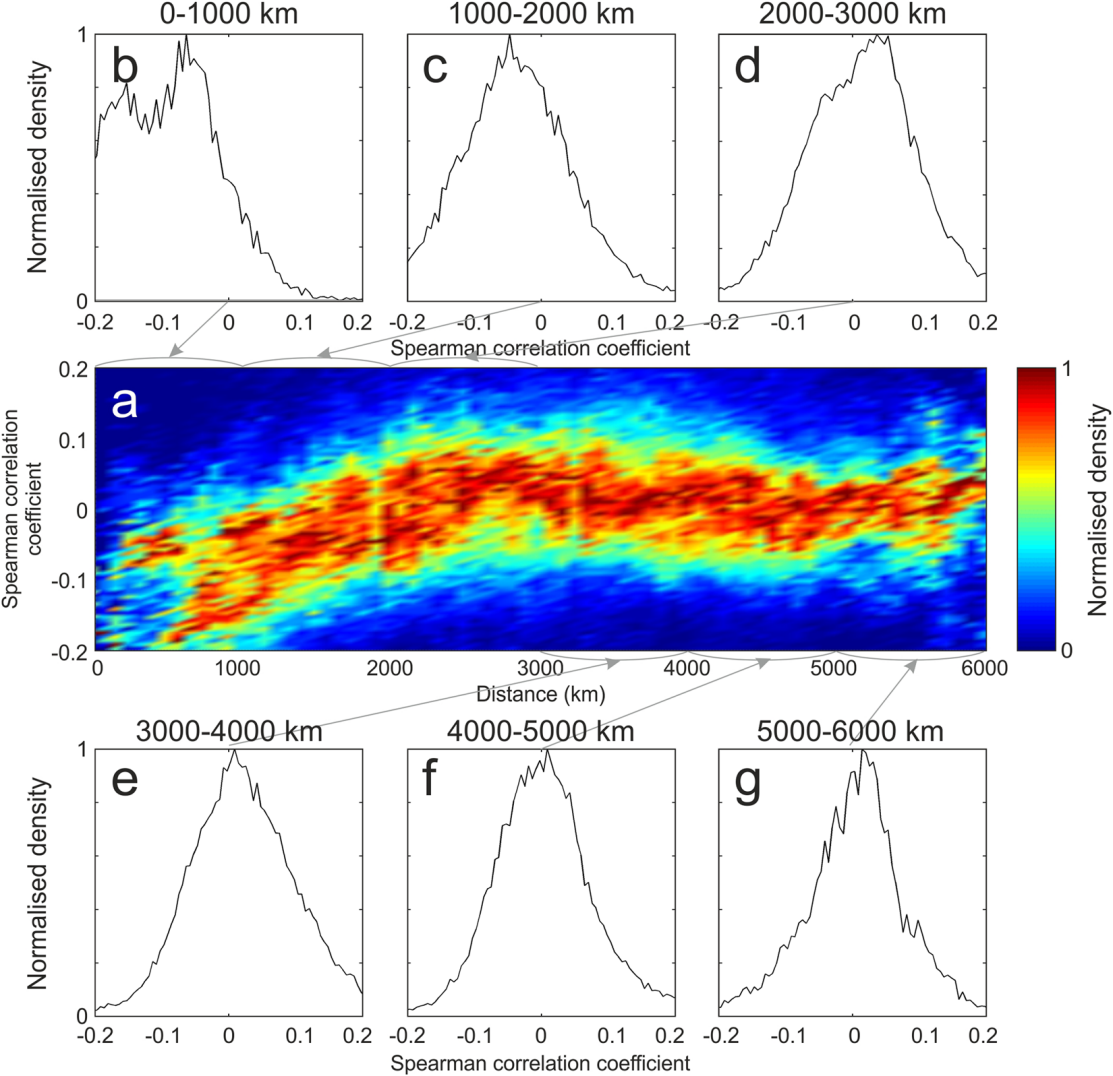
Density plots of Spearman correlation coefficient for short period secondary microseism. (**a**) 3D normalised density plot obtained for the band 2.5–5.0 s, showing the distance sea ice-seismic station in the x-axis, the Spearman correlation value in the y-axis, and the number of correlation estimations with the color scale. (**b**–**g**) 2D normalised histograms showing the Spearman correlation value in the x-axis and the number of correlation estimations in the y-axis for different distance ranges.

To verify the dependence of the anti-correlation from the distance between the sea ice and the seismic station recording microseism, a cumulative 3D density plot of all the correlation maps was obtained per each period band, showing the distance in the x-axis, the Spearman correlation value in the y-axis, and the number of Spearman correlation estimations, performed on the filtered deseasonalized time series, with the color scale (Figs 5a, S6a and S7a). These 3D density plots have the aim to highlight common patterns among the 20 maps, and reduce the contribution of features regarding single stations or a small number of stations, such as the afore mentioned apparent link between microseism and very distant sea ice. Furthermore, 2D histograms, gathering the Spearman correlation values within given ranges of distance (from 0 to 6000 km, with step of 1000 km), were obtained (Figs 5b–g, S6b–g and S7b–g). Both the 3D density plots and the 2D histograms show a fairly symmetric shape with maxima in correspondence with zero correlation values, that is what we expect in case of unrelated random signals. However, if we focus on the short distances (<1000 km; Figs 5b, S6b and S7b), the distributions show a clear asymmetry with higher number of negative correlation values with respect to the positive correlation values. This feature, evident for all the investigated period bands, suggests that microseism is mostly affected by the sea ice concentration within 1000 km from the station recording the seismic signal. Such a decrease in sea ice sensitivity of microseism, due to the increasing distance from the seismic station, has never been observed in Antarctica. In the Arctic, similar observations led^30^ to build an equation linking sea ice and microseism amplitudes in Bering Sea.

### Unravelling microseism-sea ice link by machine learning

**Figure 6:**
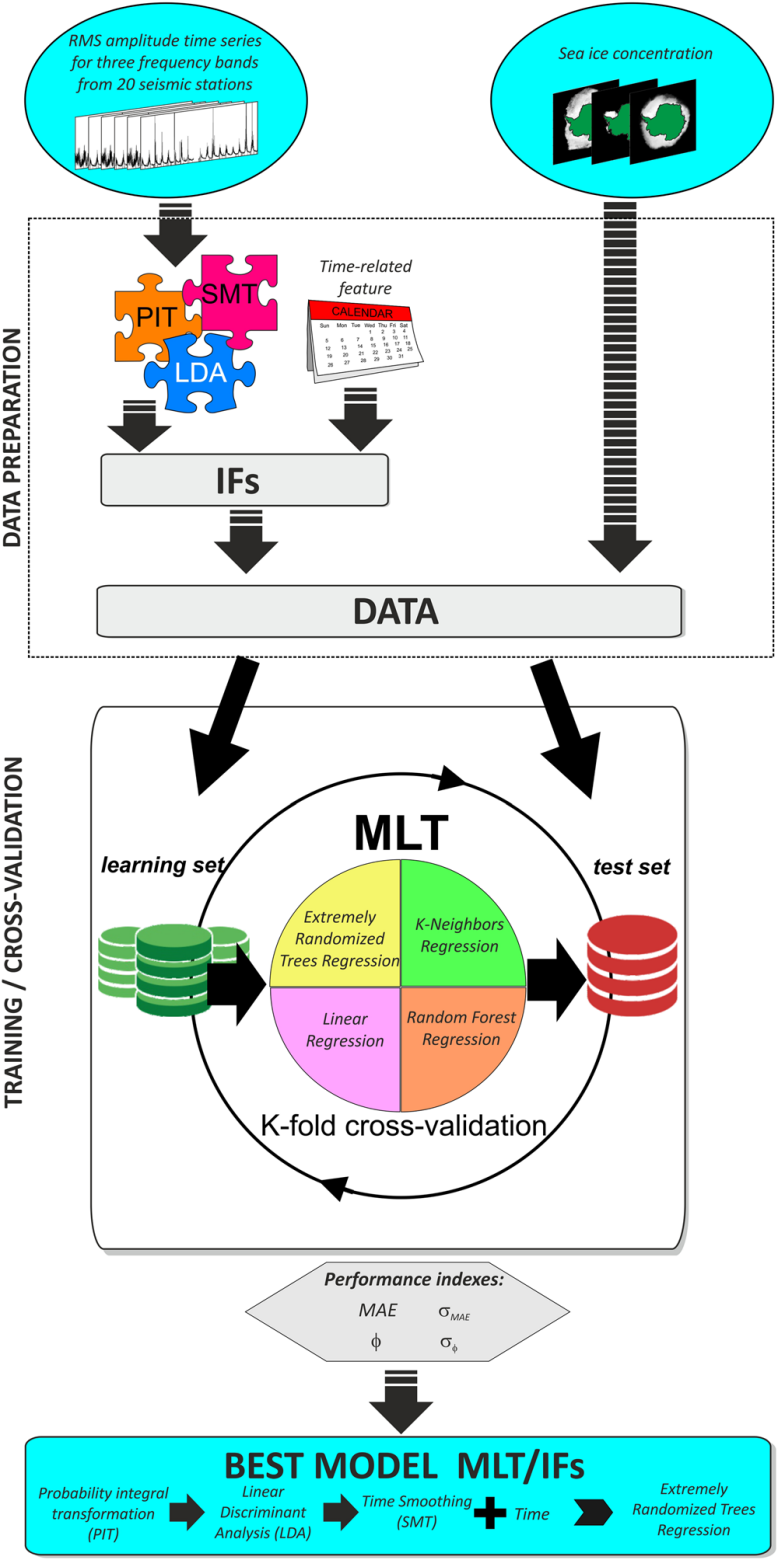
Machine learning scheme. Scheme of the modelling analysis to get the spatial distribution of sea ice concentration by using the microseism (see text for details). “IFs” stands for input features, “MLT” for machine learning technique, “PIT” for probability integral transformation, “LDA” for linear discriminant analysis, “SMT” for time smoothing, “MAE” for mean absolute error. The maps were created by Matplotlib package for Python^61^.

The observed decrease in sea ice sensitivity of microseism, due to the increasing distance from the seismic station, paves the way to implement an algorithm to spatially and temporally reconstruct the sea ice distribution around Antarctica on the basis of the microseism amplitudes. However, to do that, an analytical approach, based on microseism wave propagation, seems to be impracticable for the few and sparse data available in a highly heterogeneous and complex environment that would conduct to a strongly underdetermined ill-posed inversion problem. For this reason, we exploited the capabilities of the newest regression algorithms in machine learning to reconstruct the sea ice field starting from the knowledge of the microseism features or their transformations. In particular, the method we used is composed of three main steps (summarized in Fig. 6): (i) data preparation; (ii) training; (iii) cross-validation.

As for the step (i), to exploit the maximum information content of the microseism data, we applied the following transformations on all the RMS amplitude time series: probability integral transformation (PIT)^31^, Linear Discriminant Analysis (LDA)^32^, Time Smoothing (SMT). By using them, we obtained several potential sets of input features (hereafter referred to as IF) for machine learning modeling. In addition to the transformed microseism data, also a time-related feature, defined as a sinusoidal oscillator between 0 and 1 with annual period, and with 0 corresponding to the time with the maximum peak of the sea ice extent (defined as the measurement of the area of ocean where there is at least 15% of sea ice concentration), was introduced.

Concerning the training step (ii), we exploited the potentiality of machine learning techniques (hereafter referred to as MLTs) to build a regression model able to predict the sea ice concentration from microseism-related features. In particular, we tested the following supervised machine learning techniques: Linear Regression^33^, Random Forest Regression^34^, K-Neighbors regression^35^, and Extremely Randomized Trees Regression^36^.

Finally, regarding the last step (iii), we evaluated the unbiased generalization capacity of each pair MLT/IF by calculating the prediction performance through K-fold cross-validation^37^. The performance indexes used to compare the models were the mean absolute error (MAE) between the observed sea ice concentration and the predicted one, and the percentage (ϕ) of the cells in the sea ice grid, showing an absolute error (defined as the absolute value of the difference between predicted and true sea ice concentration) lower than 50% (threshold chosen to discriminate gross errors).

**Figure 7:**
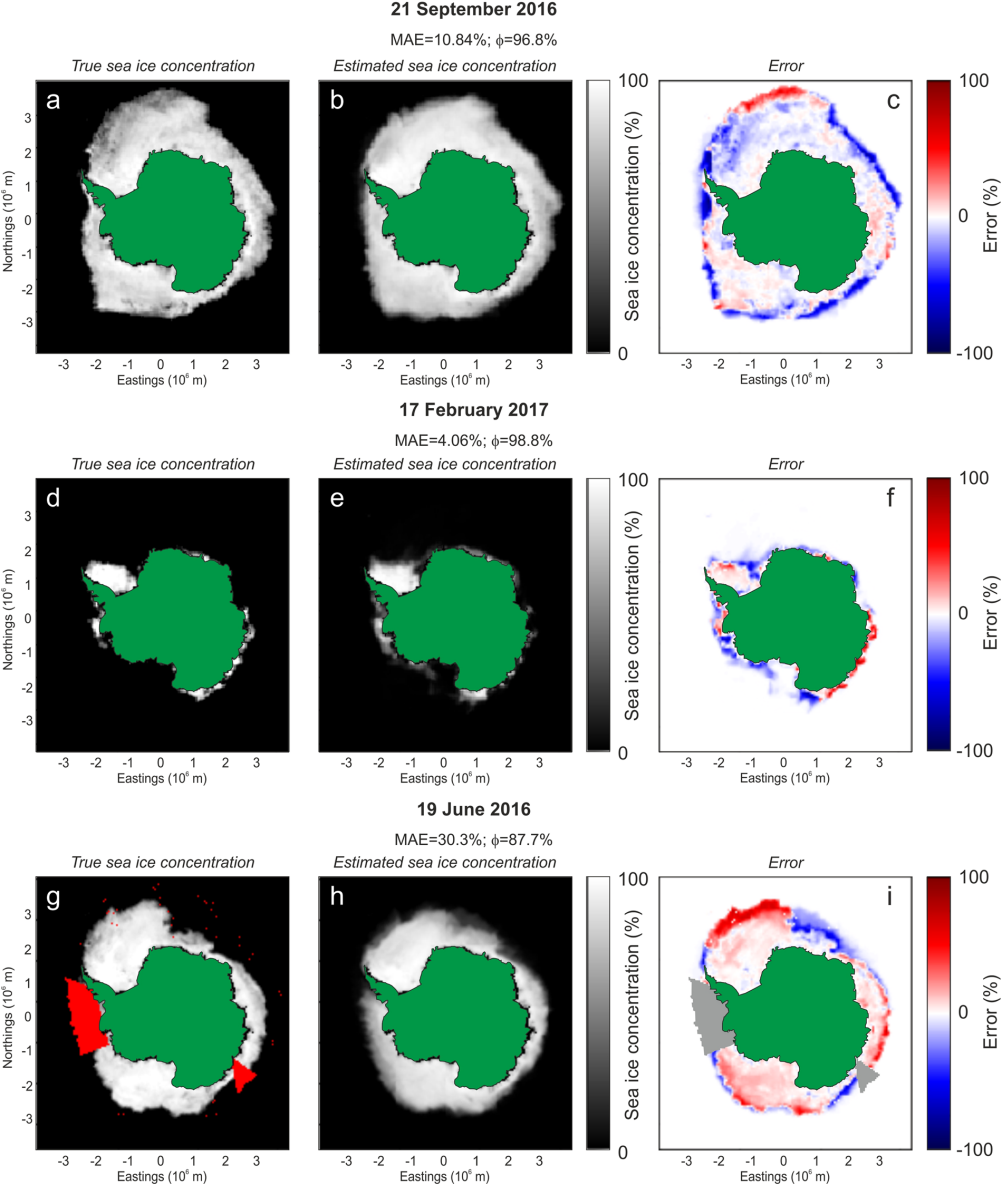
Sea ice concentration prediction. Examples of prediction based on microseism features for different patterns of sea ice concentration. In (**c**,**f**,**i**) the spatial distribution of the prediction error (computed as the difference between the true sea ice concentration and the predicted one) is plotted. The used model is obtained by Extremely Randomized Trees techniques applied on RMS amplitude data sequentially post-processed by PIT, LDA and SMT, with the addition of the time-related feature. The red areas in (**g**) and the grey areas in (**i**) represent two extended coastal regions, where the sea ice satellite data were missing on 19 June 2016. The maps were created by Matplotlib package for Python^61^.

By listing the cross-validated performances of the different pairs MLT/IF sorted by ascending MAE plus its standard deviation (*σ*_*MAE*_) (Table S1), we found that the best performance is obtained by the Extremely Randomized Trees Regression applied on RMS amplitude data sequentially post-processed by PIT, LDA and SMT, with the addition of the time-related feature. The analysis of the results also shows that the time-related feature by itself (see rows with “−” in column Basic data in Table S1) is not able to give performance comparable with those obtained with microseism features. Figure 7a–f shows two cases of actual and predicted sea ice concentration, together with the corresponding error, obtained by using the Extremely Randomized Trees model trained on the above-mentioned optimal IFs, acquired during 2011–2012 and from the beginning of 2016 to half year before the considered day.

For the identified optimal pair MLT/IF, we also estimated and mapped the unbiased spatial MAE through K-fold cross-validation (Fig. S11a). It is evident how the area characterized by the lowest prediction error is the southern part of Weddell Sea, that is shielded from the westerlies by the Antarctica Peninsula^18^ and for this reason it is characterized by almost permanent sea ice (see an example of sea ice concentration map during a period with very low sea ice extent in Fig. 7d). Such a condition of low variability of the sea ice concentration is very easy to be learnt and then predicted by the MLT. In addition, we computed the seasonal trend of the prediction error through K-fold cross-validation. The error for each day was computed as the average of the absolute values of the difference between the true sea ice concentration and the predicted one. Then, the median of all the errors concerning the same day during the year was computed to get the seasonal trend of prediction error (Fig. S11b). It is evident how the error is higher during the time periods characterised by high sea ice concentration.

The ability of the best identified model to predict the sea ice concentration could be particularly useful when the satellite data are partial and present large uncovered areas. As an example, we considered the microseism recorded on 19 June 2016, when satellite data of two extended coastal areas were missing (red areas in Fig. 7g). We trained the model with microseism data collected during 2011–2012 and predicted the sea ice concentration of that day. Figure 7h shows the predicted ice field also in the areas without satellite data coverage.

**Figure 8:**
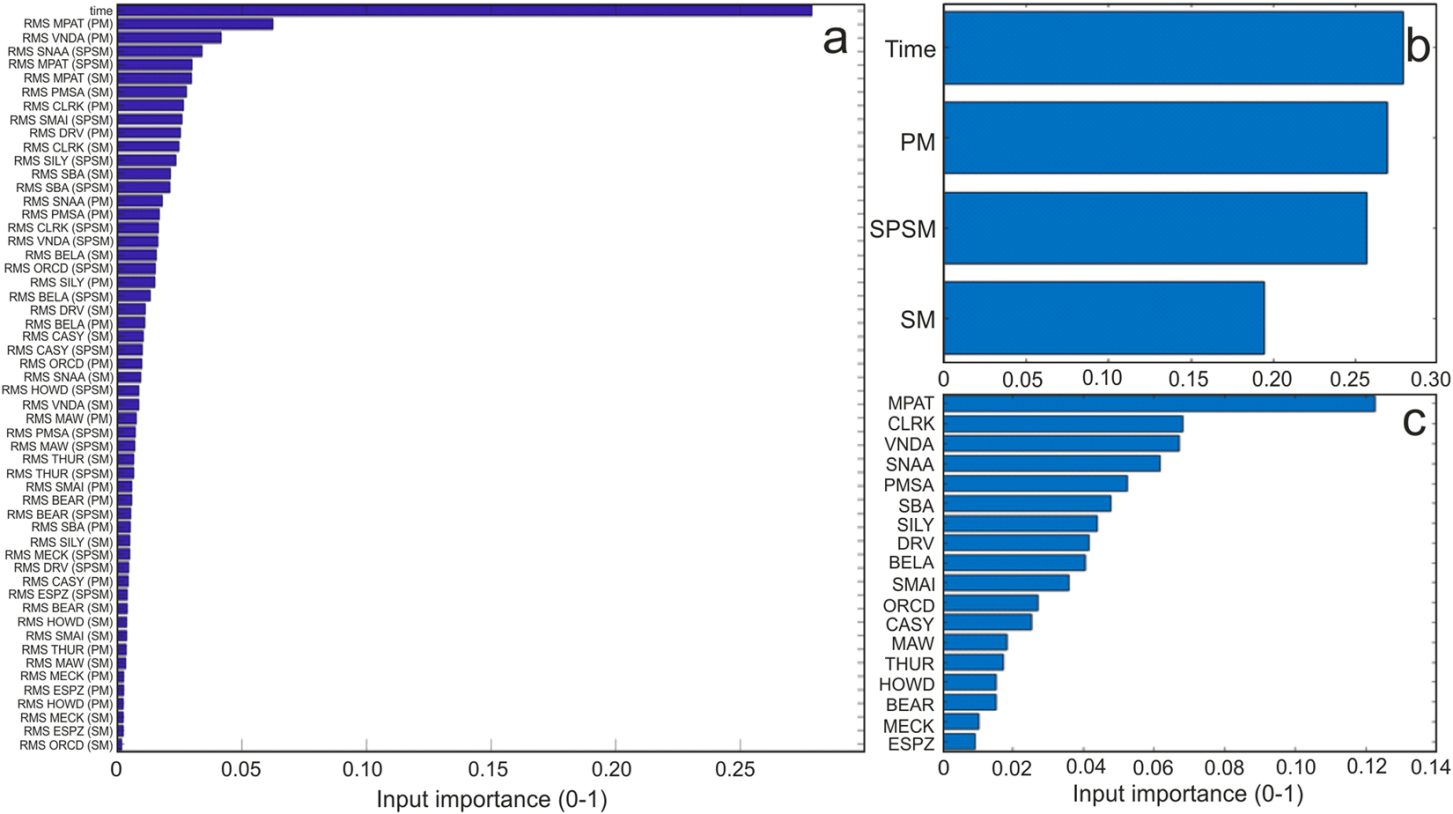
Input importance histograms. (**a**) Index of importance for all the input taken into account. Aggregation through summation of the input importance allowing to rank the microseism bands (**b**) and the seismic station (**c**) for sea ice concentration reconstruction purposes. “PM”, “SM”, “SPSM” and “time” in the y-label of (**a**,**b**) indicate primary microseism, secondary microseism, short-period secondary microseism and the time-related feature, respectively.

The Extremely Randomized Trees approach has the advantage to easily supply an index of input importance (Fig. 8). Even if the time-related feature shows a fairly high importance score (~0.28), due to the seasonality of sea ice concentration, the sum of the importance of the other microseism-related features is much higher (~0.72). This demonstrates that microseism data carry much more information about sea ice concentration than the simple seasonality. Concerning microseism, the primary microseism data and the short-period secondary microseism data roughly share the same importance for reconstructing the sea ice field, while the secondary microseism shows a slightly smaller contribution to the same purpose (Fig. 8a,b). This observation strongly supports the mostly near‐coastal origin of the microseism in primary and short-period secondary bands, as evidenced by other authors^8,16,38^. Conversely, secondary microseism shows the weakest link with sea ice, testifying that its source is likely also influenced by wave–wave interaction in deep ocean, as supposed by previous authors^39,40^. Moreover, the importance of each of the 18 stations, used in this analysis, was evaluated (Fig. 8c). One of the factors that seem to mostly affect the station importance is the temporal variability of sea ice extent in the coast nearby the station. In particular, we estimated the linear extent of the sea ice, with direction approximately normal to the coastline, closest to the station during two time periods, September 2014 and March 2017, characterized by the maximum and minimum sea ice extent during the investigated time intervals, respectively (Fig. S12a–c). As shown in Fig. S12d, there is a positive correlation between the station importance and the difference of the two linear sea ice extents.

It has to be underlined that, since microseism can have distant sources unrelated to Antarctic nearshore dynamics (hence theoretically not affected by the sea ice presence)^41,42^, bias in the microseism-retrieved space-time distribution of sea ice can occur in case of strong distant microseism sources linked for instance to distant storms. A further issue, highlighted also by^30^, could be related to the fact that sea ice concentration is assumed to be directly linked to the sea ice strength and then to the decrease in the efficiency of energy transfer from ocean to solid earth. Such an assumption could be not entirely verified. In spite of these issues, the prediction capability of the proposed model is satisfying as testified by the MAE value equal to 10.3%, obtained by the optimal pair MLT/IF.

## Discussion

In summary, we quantitatively investigated the relationship between microseism recorded along the coasts of Antarctica and the sea ice concentration in the whole Southern Ocean.

Clear anti-correlation patterns between microseism and sea ice concentration were found at all the investigated microseism bands (Figs 4 and S3). Such a relationship depends on the fact that microseism amplitudes are influenced by ocean wave heights^6,19,20^. Indeed, if we assume that sea ice concentration is a proxy of sea ice strength^30^, the increase in sea ice concentration: (i) prevents swell from reaching the coast, decreasing the efficiency of primary microseism generation and (ii) inhibits the swell reflection along the coast, reducing the secondary microseism energy^15,16^. In particular, as for the short-period secondary microseism, such a band is likely to be generated by local nearshore wave-wave interaction^7^, heavily modulated by the presence of sea ice.

The microseism bands, showing the strongest link with sea ice, are primary and short-period secondary microseism (Fig. 8b), corroborating their mostly near‐coastal origin. On the other hand, the weakest link identified between sea ice concentration and secondary microseism (Fig. 8b) is indicative of influences by wave–wave interaction in deep ocean on the secondary microseism source.

In addition, we clearly show a decrease in sea ice sensitivity of microseism, due to the increasing distance from the station recording the seismic signal. The influence seems to disappear for distances above 1,000 km (Figs 5, S6 and S7). Following the reasoning of^30^, such a distance could be related to the attenuation length. Indeed, taking into account the entire period band of microseism (from 20 s to 2.5 s), attenuation coefficient of ~10^−3^ km^−1^ can be found in literature^43,44^. Hence, the corresponding attenuation lengths turn out to have the same order of magnitude as the estimated maximum distance of influence of sea ice on microseism. Moreover, this distance could also be linked to the temporal variability of sea ice extent in the coast nearby the station. Indeed, the average value of the difference between maximum and minimum linear extent of the sea ice, measured approximately normal to the coastline closest to the station during September 2014 and March 2017 (characterized by the maximum and minimum sea ice extent during the investigated time intervals, respectively; Fig. S12b,c), is equal to 850 km (average of the x-axis values of Fig. S12d), and then very similar to the estimated maximum distance of influence of sea ice on microseism. Furthermore, this 1,000 km-distance does not seem to be related to the bathymetry. Indeed, focusing on primary microseism at period of 20 s and following the Airy linear wave theory approximation, most primary microseism generation should occur at water depths less than ~150 m^20^. As for the short period secondary microseism (the other microseism type showing the strongest link with sea ice), its sources are mostly located at even shallower depths (e.g.^8,45^). If we consider the 150 m depth, this limit corresponds to distances from the Antarctic coastline much shorter than 1,000 km^46^.

It is worth noting that such a 1,000 km-threshold, that has to be considered as an average value among the different stations, does not signify that microseism recorded in Antarctica cannot have also distant sources, as highlighted by several authors (e.g.^18,26,41,42^). This study does not constrain the locations of the microseism sources, their amplitudes and the corresponding decay with distance, but rather suggests that sea ice with maximum distance of 1,000 km, on average, contributes to modulate the microseism amplitude. Indeed, location of microseism sources is a hard task, as microseism signals are non-impulsive, and the sources are generally diffuse and variable in time^18^. Hence, the classical location algorithms, used in earthquake seismology and based on the picking of the different seismic phases, cannot be applied to locate microseism sources. Array processing techniques, that can overcome the above-mentioned difficulties, have provided locations of microseism source areas surrounding Antarctica^26^. However, as the array data available in Antarctica are sparse, the microseism array locations have been obtained only for short time intervals (for instance a couple of months in^26^). Preliminary information about the direction of the microseism sources, with less accuracy than by using array data, can also come from polarization analysis of single 3-component station^25^.

The microseism sensitivity in sea ice is reflected in the slightly different annual patterns of RMS amplitude time series observed among the stations (Fig. S2). Such differences can be partly interpreted as due to the different sector, where the stations are located. Indeed, as stated by^47^ there are regional changes in the annual cycle of sea ice extent in the five Antarctic sectors. For instance, the least sea ice cover observed in the West Pacific sector, compared to the other sectors, justifies the shorter duration of the time interval characterised by minimum RMS amplitudes (particularly evident in CASY). In addition, it is also possible to observe peculiar patterns of specific stations, such as ESPZ and ORCD. For these two stations, the duration of the time interval characterised by microseism RMS amplitude minima is shorter compared to the others, likely reflecting the shorter-lived effect of sea ice modulation on microseism at the relatively lower latitudes of ESPZ and ORCD.

Finally, we propose an innovative method, based on up-to-date machine learning techniques, able to reconstruct the spatial-temporal distribution of sea ice concentration by using microseism recorded in different period bands by distinct seismic stations. The importance of each station in the prediction of sea ice concentration was evaluated (Fig. 8c) and turned out to be mostly affected by the temporal variability of sea ice extent in the coasts nearby the station (Fig. S12). Hence, the wider the area, close to the station and characterised by intense sea ice time variability, the stronger the modulation effect on the microseism amplitude recorded by the station, and then the higher the station importance for sea ice concentration prediction.

The quality of the modelling results, obtained by the machine learning techniques for the relatively small dimension of measured data, indicates that microseism signal carries significant information about the surrounding sea ice concentration. This technique will allow reconstructing the sea ice concentration in both Arctic and Antarctica in periods when the satellite images, routinely used for sea ice monitoring^27^, are not available, with wide applications in many fields, first of all the climate studies.

A future development of this study will be the inclusion of time series of horizontal seismic component amplitudes as input in the machine learning modelling. While the vertical component mainly brings information about Rayleigh waves composing microseism, the horizontal components allow taking into account both Rayleigh and Love waves. Indeed, it has been highlighted by recent papers that the contribution of Love waves in microseism can be significant^45,48^. In the light of this, also the wavefield features (quantified by the polarization parameters, i.e. incidence angle, azimuth angle, rectilinearity, planarity)^49^ could add additional information to the machine learning modelling, if included as further inputs. The inclusion of these new inputs will likely improve the capability to reconstruct the spatial-temporal distribution of sea ice concentration around Antarctica by the microseism.

## Methods

### Spectral and RMS amplitude analyses

The Short Time Fourier Transform (STFT) of the data recorded by the vertical component of the 20 stations shown in Fig. 1 was calculated as follows: spectra over 81.92-second-long sliding window were computed, and all the spectra falling in the same day were averaged by Welch’s overlapped segment averaging estimator^50^, and visualised as spectrograms (Fig. 2).

As for the root mean square (RMS) amplitudes, three distinct period bands were taken into account: primary microseism (13–20 s), secondary microseism (5–10 s), and short period secondary microseism (2.5–5.0 s)^18^. The RMS amplitude time series were obtained, gathering median daily RMS amplitudes, computed on values calculated over consecutive 81.92-sec-long windows (Fig. S1).

Successively, the RMS amplitude time series were smoothed by a 90-day-long moving median, split in year-long windows, that were stacked and normalised by subtracting the minimum value and dividing by the maximum value (Fig. S2a). Then, all these stacked normalised RMS amplitudes of all the stations were again stacked to have the overall seasonal trends in the three distinct period bands (Fig. S2b). Finally, the three curves were stacked, thus obtaining a single curve (Fig. S2c). Hence, the highest and lowest peaks in Fig. S2c indicate the onset time of the 90-day-long windows, characterised by the strongest (February–April) and weakest (October–December) microseism, respectively.

Furthermore, a median value of RMS amplitude was obtained per each station in the three considered period bands and, by applying a triangulation-based natural neighbour interpolation^51^, a map showing the spatial distribution of RMS amplitude values was plotted per each period band (Fig. 3a–c). Following^52^, to remove interpolated information from poorly constrained regions, we masked interpolated data for areas farther than 1000 km from any station (distance chosen to evidence the unreliable interpolated microseism amplitude information in the inner part of Antarctica). The same technique was applied to generate maps focused on the periods with the maximum (February–April; Fig. 3d–f) and minimum (October–December; Fig. 3g–i) microseism amplitudes.

### Correlation analysis

Following the idea by^18^, we quantitatively investigated the relationship between microseism and sea ice distribution. Time series of daily sea ice concentration values, defined as the percentage of ice cover within each grid node (cell size of 25 × 25 km^2^), were obtained and compared with the RMS amplitude patterns by Spearman correlation coefficient. This coefficient, defined as a nonparametric measure of rank correlation, was preferred with respect to the more widely used cross correlation coefficient, whose application is limited to explore linear dependence between normally distributed parameters^53,54^. Then, spatial distributions of the Spearman correlation coefficient in the portion of Southern Ocean, seasonally covered by sea ice, were obtained (Figs 4a and S3a,c).

We applied the same method on RMS amplitude and sea ice time series, where the long-period components were filtered out. To obtain these filtered time series, we tested two methods: (i) we computed smoothed time series of RMS amplitude and sea ice by using a 15-day-long moving median, and subtracted from each value of the original RMS amplitude and sea ice series, the value of the smoothed series shifted back by one year; (ii) we divided the RMS amplitude and sea ice time series into 1-year-long windows, stacked them and smoothed them by using a 15-day-long moving median, and then we subtracted from each sample of the original time series the corresponding sample of the yearly stacked series. By using both methods, we obtained very similar time series. Also in this case, we obtained maps of spatial distribution of Spearman correlation coefficient (Figs 4b and S3b,d).

To test whether the observed values of Spearman correlation coefficient are significantly different from zero or not (null hypothesis), the t-test, taking into account the different number of samples in the distinct RMS amplitude time series, was performed and the space distribution of p-value (probability value) was calculated; in particular, p-values lower than the significance level of 0.05 were considered sufficient to reject the null hypothesis (Fig. S4). Such a threshold means that the probability, that the result of the statistical test is due to chance alone, is less than 5%, so it would occur once out of 20 times the study is repeated. The value of 0.05 is a commonly accepted significance level used for this statistic test (e.g.^18^).

Focusing on the filtered RMS amplitude and sea ice time series, we collected all the Spearman correlation values and the corresponding distances for all the stations and the three period bands. A cumulative 3D density plot was obtained per each period band, showing the distance in the x-axis, the Spearman correlation value in the y-axis, and the normalised number of Spearman correlation estimations with the color scale (Figs 5a, S6a and S7a). Furthermore, 2D histograms, gathering the Spearman correlation values within given ranges of distance (from 0 to 6000 km, with step of 125, 250, 500, 1000 km, see Figs 5 and S6–S10), were obtained. Figures 5b–g, S6b–g and S7b–g show the results for the 1000-km step.

### Calculation of sea ice concentration by using microseism and machine learning

The method is composed of three steps (Fig. 6): (i) data preparation; (ii) training; (iii) cross-validation.

As for the step (i), the time intervals characterized by an almost complete coverage of seismic data from 18 out of 20 stations were selected (2011–2012 and 2016–2017), and the corresponding RMS amplitude data were extracted. SYO and DNTW stations were not used because of the total lack of data during the most recent time interval (2016–2017). We applied the following transformations on all the RMS amplitude time series:

- Probability Integral Transformation (PIT^31^): to maximize the distribution regularity of the data in a range. The technique attempts to convert a random variable with any given continuous distribution into a random variable having a standard uniform distribution^55^.
- The Linear Discriminant Analysis (LDA^32^): to maximize the class-separability of clustered data. The goal of LDA is to project the dataset onto a lower-dimensional space with better class-separability. To define the classes of sea ice space distribution, we firstly found the best number of clusters that can separate the sea ice space distribution images by using the Calinski-Harabasz^56^ and the Silhouette^57^ indexes in the range between 2 and 365. We found the optimum number of 32 different classes for the sea ice space distribution dataset, that we clusterized by k-means algorithm^58^. Then, we applied the LDA to the seismic features corresponding to these classes. With an explained variance greater than 95%, we found the transformed and reduced seismic features that better separate the sea ice space distribution classes.
- Time Smoothing (SMT): to reduce the time variability of the seismic data, we applied a sliding window smoothing the signal. The mean autocorrelation function of the RMS amplitude time series shows a fast decay of more than 60% after only 2 days, thus we considered a causal-consistent smoothing window of 3 days.

As potential input features (IF) for machine learning modeling, we tested all the possible ordered selections without repetition of any subset (included the empty one) of the described transformations (PIT, LDA, SMT) on the RMS amplitude data. To be sure that the model predicted the sea ice concentration from the information carried by microseism signals and not from the implicit seasonality, we compared the results obtained by using only a variable linked to the period of the year as input (see rows with “−” in the Basic data column of Table S1) with those obtained by the whole set of microseism-related data (see rows with “microseism” in the Basic data column of Table S1). The time-related feature was defined as a sinusoidal oscillator between 0 and 1 with annual period and with 0 corresponding to the time with the maximum peak of the sea ice concentration. All the potential sets of IFs were tested both in presence (see rows with “x” in the Time feature column of Table S1) and in absence of this time-related feature that indicated the day of the year (see rows without “x” in the Time feature column of Table S1).

Concerning the training step (ii), the supervised machine learning techniques (MLT), taken into account to predict the sea ice concentration, were:

- Linear Regression^33^: the relationships are modeled using linear predictor functions, whose unknown model parameters are estimated from the input/output data.
- Random Forest Regression^34^: it operates by constructing a multitude of decision trees at training time, outputting the mean prediction of the individual trees.
- K-Neighbors Regression^35^: average of the values of its k nearest neighbors.
- Extremely Randomized Trees Regression^36^: based on random forest, it applies a fully random selection to split data in the test nodes.

The step (iii), cross-validation, consisted of evaluating the unbiased generalization capacity of each pair MLT/IF by calculating the prediction performance through K-fold cross-validation^37^. We considered K = 10 with partially overlapped subsets of 365 timely-consecutive daily data. The choice to consider 365-consecutive daily data for testing was due to the slow dynamics of sea ice formation and melting. In a completely random selection of the test set, results could be optimistically biased for the presence of close-in-time (and hence similar) patterns in the learning set. Considering an annual (i.e. 365 daily data) test set, we avoided biases in results due to such seasonal correlations. The performance indexes used to compare the models were:

- The mean absolute error (MAE) between the observed sea ice concentration and the predicted one, calculated in a mask where the ice concentration is not null for the whole period.
- The percentage (ϕ) of the cells in the sea ice grid, showing an absolute error, defined as the absolute value of the difference between predicted and true sea ice concentration, lower than 50% (threshold chosen to discriminate gross errors).

Cross-validation allowed us to estimate the unbiased mean and standard deviation (σ) of the performance indexes. Table S1 shows the cross-validated performances of the different pairs MLT/IF sorted by ascending *MAE* + *σ*_*MAE*_. The best performance was obtained by using the technique of Extremely Randomized Trees on RMS data sequentially post-processed by PIT, LDA and SMT with the addition of the time-related feature.

The Extremely Randomized Trees approach has the advantage to easily supply an index of input importance^59^. However, the optimal model uses the LDA data transformation which projects the original data into a new linear space. Hence, to reconstruct the importance of the original inputs, we had to back-project the importance of the surrogate features to the original space through the normalized eigenvectors used in the transformation (Fig. 8a). Figure 8b,c aggregates (through summation) the importance of single inputs by their classes of microseism bands and seismic stations, respectively.

## Supplementary information

Supplementary figures and table

## Data Availability

The facilities of IRIS Data Services, and specifically the IRIS Data Management Center, were used for access to waveforms of 20 seismic stations (https://ds.iris.edu/SeismiQuery/station.htm). IRIS Data Services are funded through the Seismological Facilities for the Advancement of Geoscience and EarthScope (SAGE) Proposal of the National Science Foundation under Cooperative Agreement EAR-1261681. In particular, we used data from Antarctic Seismographic Argentinean Italian Network (BELA, ESPZ, ORCD, SMAI), POLENET (BEAR, CLRK, DNTW, HOWD, MECK, MPAT, SILY, THUR), Global Seismograph Network (CASY, PMSA, SBA, VNDA), GEOSCOPE (DRV), Geoscience Australia (MAW), GEOFON (SNAA), Pacific21 (SYO). Information about temporal and spatial variability of sea ice concentration were downloaded from the website https://nsidc.org/data/g02135. Distribution of the data set from NSIDC is supported by the NOAA@NSIDC Team with funding from NOAA and with assistance from the NSIDC NASA DAAC. This site is maintained with assistance from the NSIDC NASA DAAC. Figure 1 was created by using Antarctic Mapping Tools for MATLAB®^60^. The maps in Figs 6, 7 and S11 were created by Matplotlib package for Python^61^.
