# Supplementary material for: Exploring the link between microseism and sea ice in Antarctica by using machine learning
Source: Sci Rep. 2019 Sep 10;9:13050. doi: 10.1038/s41598-019-49586-z (PMC6736882; doi:10.1038/s41598-019-49586-z)
Supplement: Supplementary file 1 — Supplementary figures and table [file 41598_2019_49586_MOESM1_ESM.docx]

Exploring the link between microseism and sea ice in Antarctica by using machine learning

Andrea Cannata^1,2*^, Flavio Cannavò^2^, Salvatore Moschella^1^, Stefano Gresta^1^, and Laura Spina^3^

1. Università degli Studi di Catania, Dipartimento di Scienze Biologiche, Geologiche e Ambientali - Sezione di Scienze della Terra, Corso Italia 57, I-95129, Catania, Italy.
2. Istituto Nazionale di Geofisica e Vulcanologia, Osservatorio Etneo – Sezione di Catania, Piazza Roma 2, 95125 Catania, Italy.
3. Istituto Nazionale di Geofisica e Vulcanologia, Sezione di Roma 1, Via di Vigna Murata, 605, 00143 Roma RM, Italy.

******* *Corresponding Author:*

Dipartimento di Scienze Biologiche, Geologiche e Ambientali

Università degli Studi di Catania

Corso Italia 57

95125 Catania (Italy)

phone number: +39 335 6474 932

email: [andrea.cannata@ingv.it](mailto:andrea.cannata@ingv.it)

Supplementary Materials


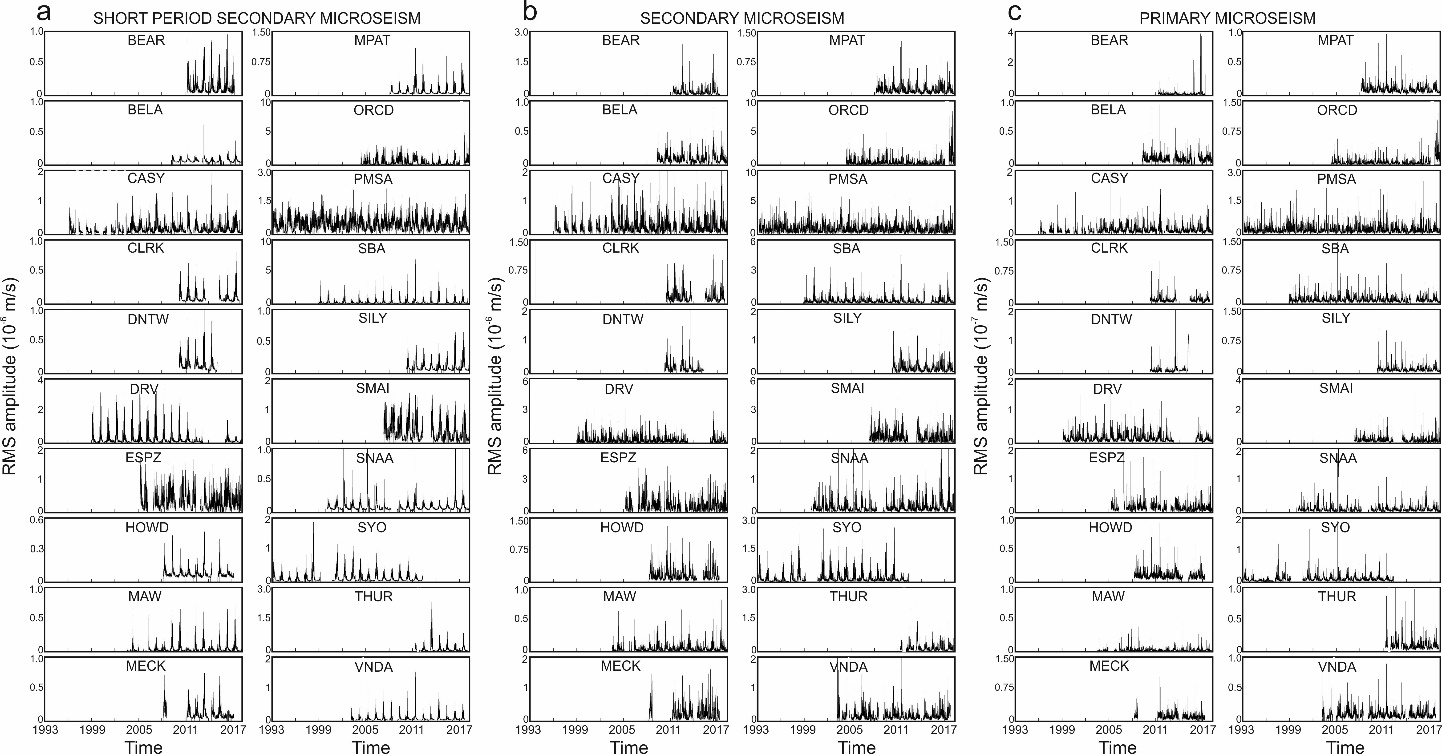


Fig. S1. RMS amplitude time series. RMS amplitudes of the seismic signal recorded by the vertical component of the 20 considered stations, and filtered in the band (a) 2.5-5.0 s (short period secondary microseism), (b) 5-10 s (secondary microseism) and (c) 13-20 s (primary microseism).

**
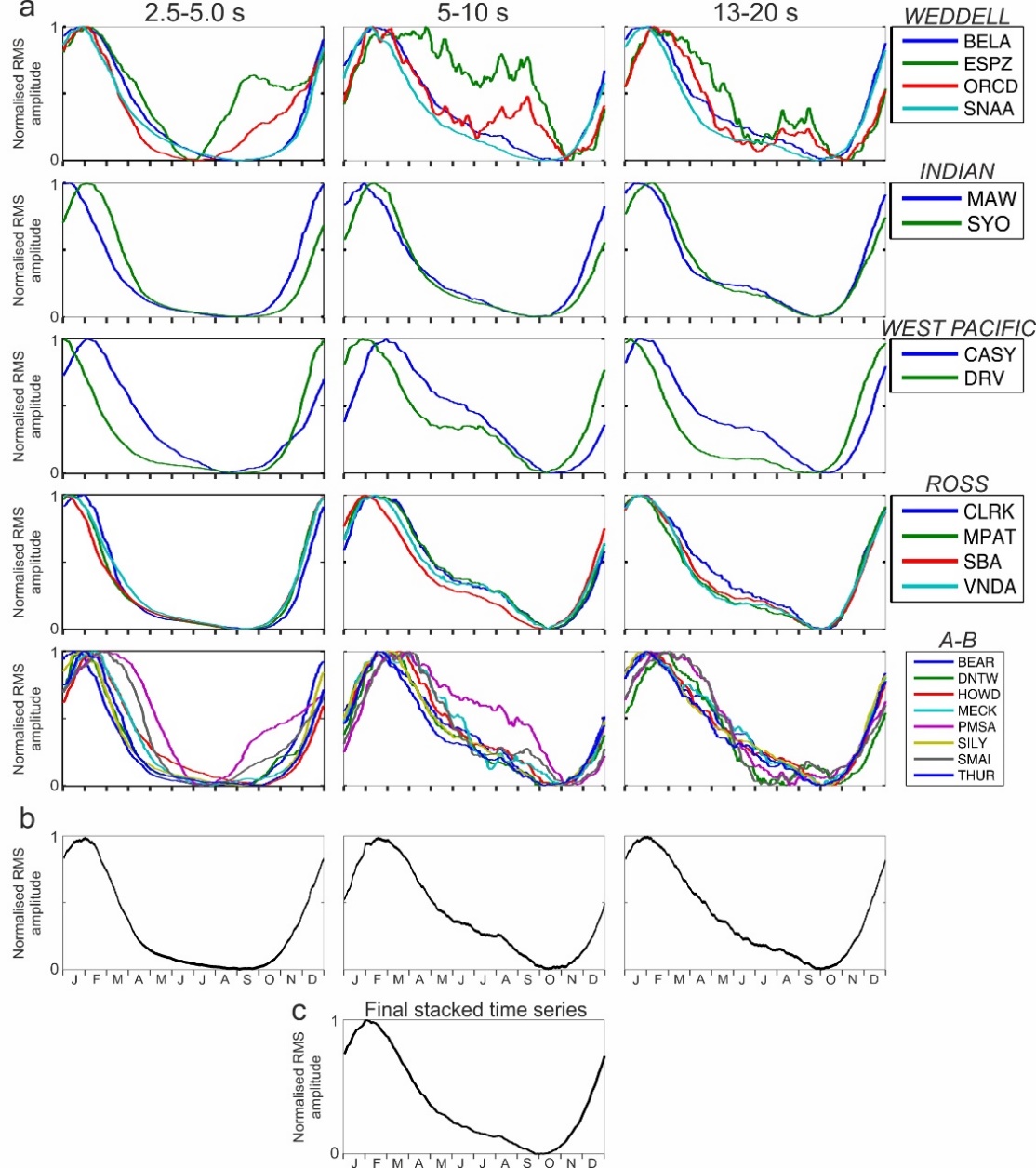
**

**Fig. S2. Stacked RMS amplitude time series.** (a) RMS amplitude time series, smoothed by a 90-day-long moving median, split in year-long windows, stacked and normalised for all the considered seismic stations (see the legends on the right). In particular, left plots regard the period band 2.5-5 s, central plots 5-10 s and right plots 13-20 s. The time series are gathered in different plots, according to the different Antarctic sector, where the station is located (see **Figure 1** for sector view). (b) Overall seasonal trend of microseism for the period band 2.5-5 s, 5-10 s and 13-20 s (left, central and right plot, respectively), obtained by stacking the RMS amplitudes of all the stations, smoothed and normalised, shown in (a). (c) Overall seasonal trend of microseism in Antarctica obtained by stacking the time series in (b). The time in x-axis in (a-c) indicates the window onset of the 90-day-long moving median.

**
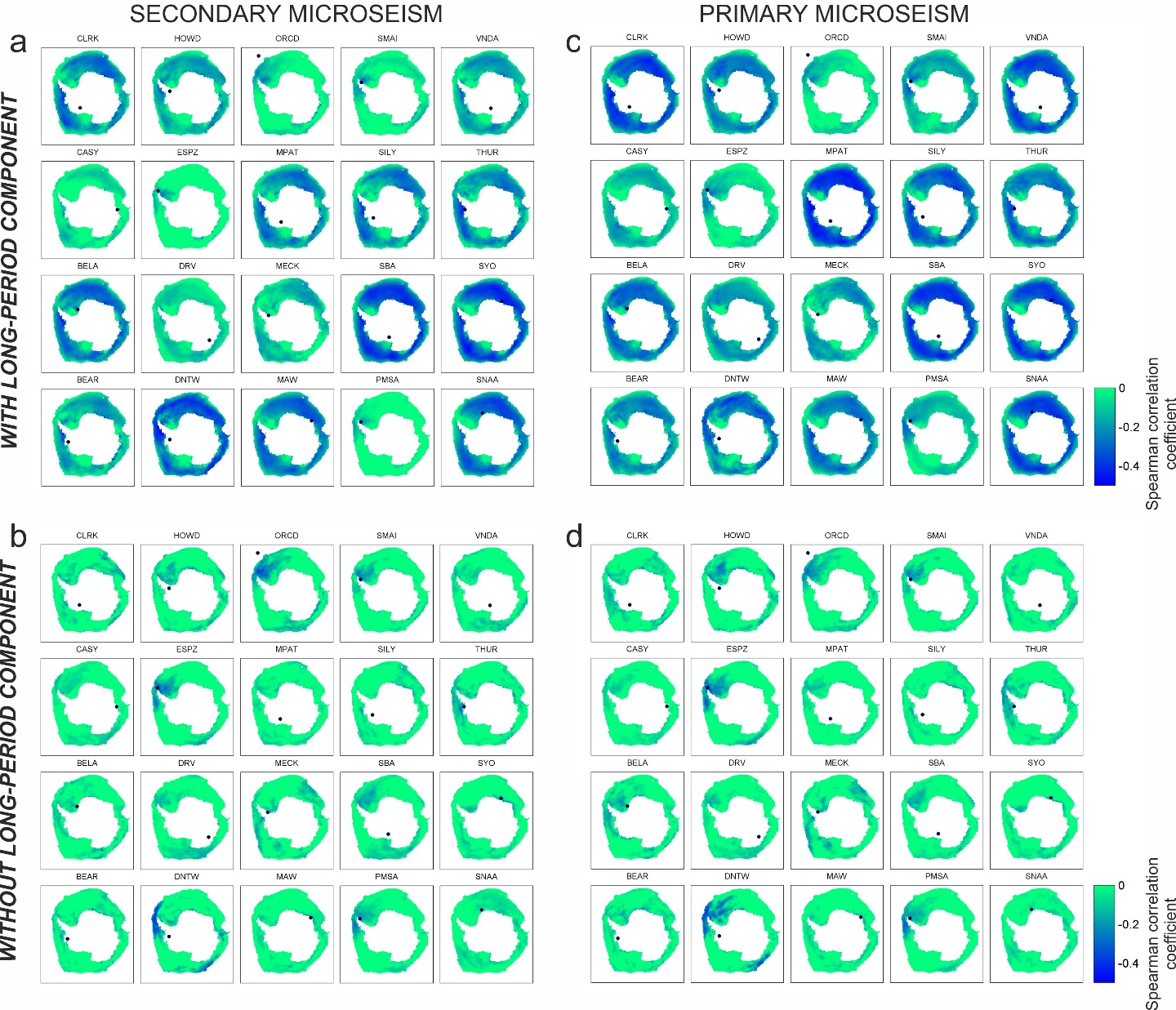
**

**Fig. S3. Spearman correlation coefficient maps for secondary and primary microseism**. Maps of Antarctica showing the space distribution of the Spearman correlation coefficient computed between sea ice concentration time series and RMS amplitudes, calculated per each considered station in the band 5-10 s (a,b) and 13-20 s (c,d). In (a,c) the long-period components of the time series were present, in (b,d) the long-period components were filtered out before performing the correlation analysis. The black dots indicate the locations of the considered stations.


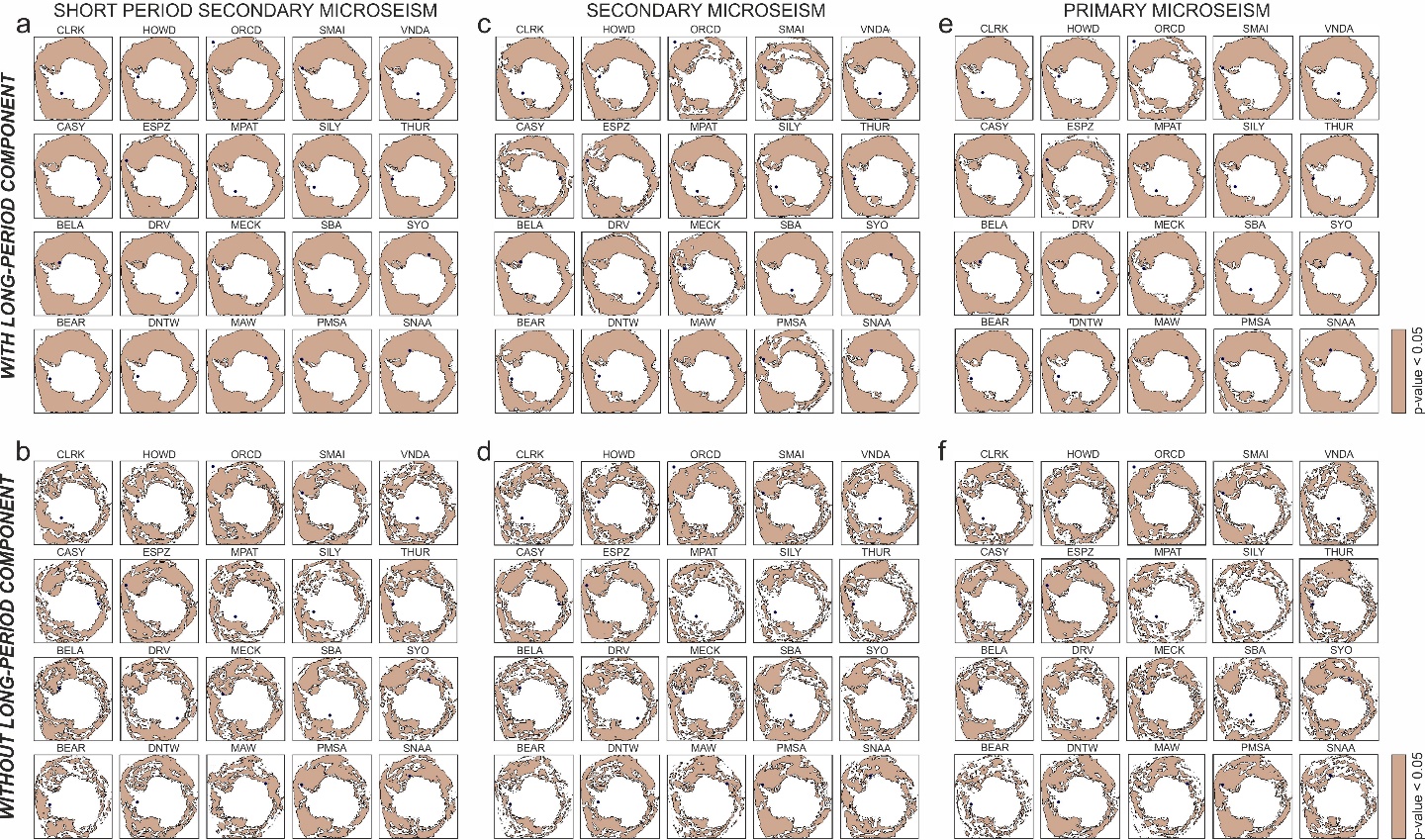


**Fig. S4*.* Maps of p-values**. Maps showing the space distribution of p-values, obtained by the Spearman correlation analysis, calculated per each considered station in the bands (a,b) 2.5-5.0 s, (c,d) 5-10 s and (e,f) 13-20 s. The pink color indicates the areas where p-value is lower than 0.05. In (a,c,e) the long-period components of the time series were present, in (b,d,f) the long-period components were filtered out before performing the correlation analysis. The black dots indicate the locations of the considered stations.


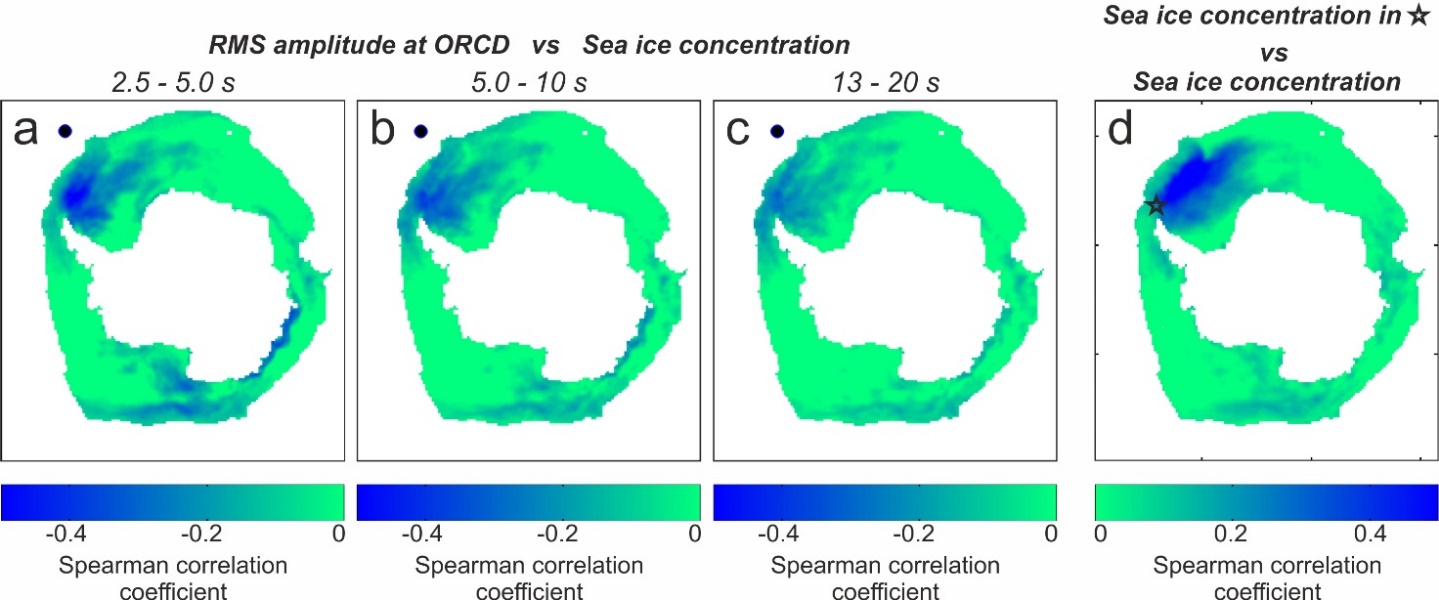


**Fig. S5.** **Spearman correlation coefficient maps of ORCD station.** (a,b,c) Maps of Antarctica showing the space distribution of the Spearman correlation coefficient computed between sea ice concentration time series and RMS amplitudes, calculated for ORCD station (black dot) in the band 2.5-5.0 s, 5-10 s and 13-20 s, respectively. (d) Map of Antarctica showing the space distribution of the Spearman correlation coefficient computed between the sea ice concentration time series in the star location and all the other time series of sea ice concentration in the whole Southern Ocean.

***
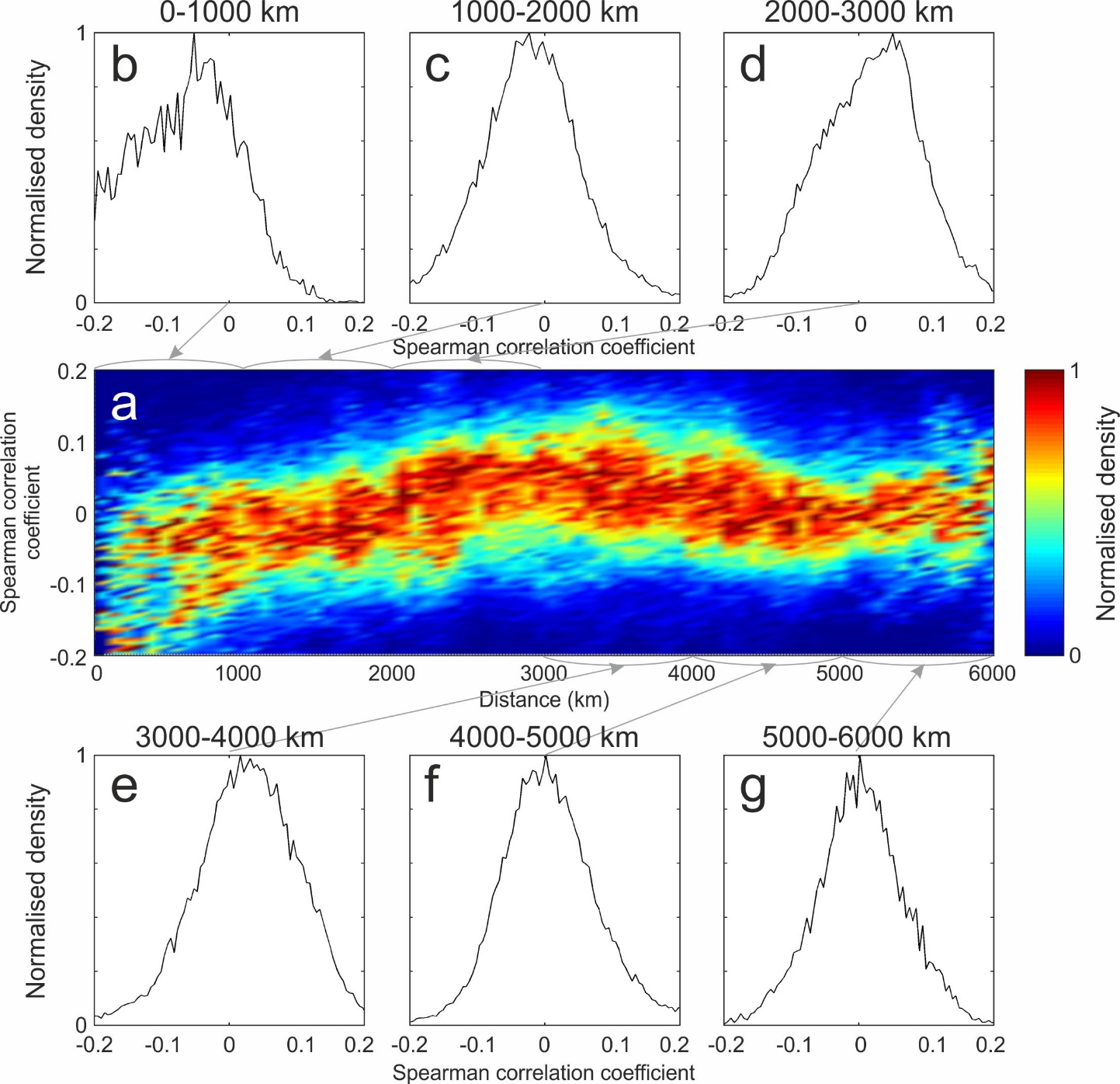
***

**Fig. S6*.* Density plots of Spearman correlation coefficient for secondary microseism.** (a) 3D normalised density plot obtained for the band 5-10 s, showing the distance sea ice-seismic station in the x-axis, the Spearman correlation value in the y-axis, and the number of correlation estimations with the color scale. (b-g) 2D normalised histograms showing the Spearman correlation value in the x-axis and the number of correlation estimations in the y-axis for different distance ranges.

***
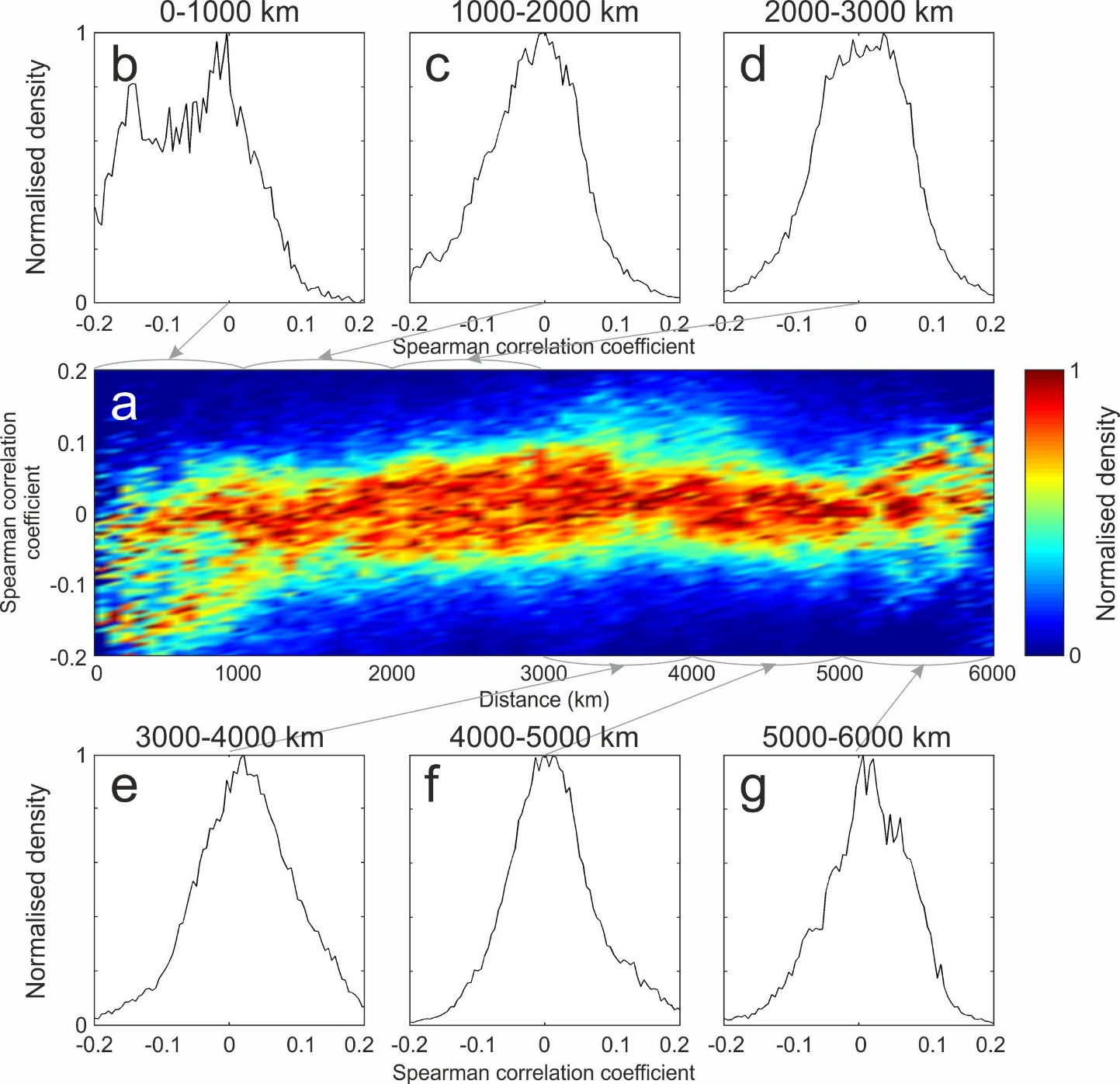
***

**Fig. S7*.* Density plots of Spearman correlation coefficient for primary microseism.** (a) 3D normalised density plot obtained for the band 13-20 s, showing the distance sea ice-seismic station in the x-axis, the Spearman correlation value in the y-axis, and the number of correlation estimations with the color scale. (b-g) 2D normalised histograms showing the Spearman correlation value in the x-axis and the number of correlation estimations in the y-axis for different distance ranges.


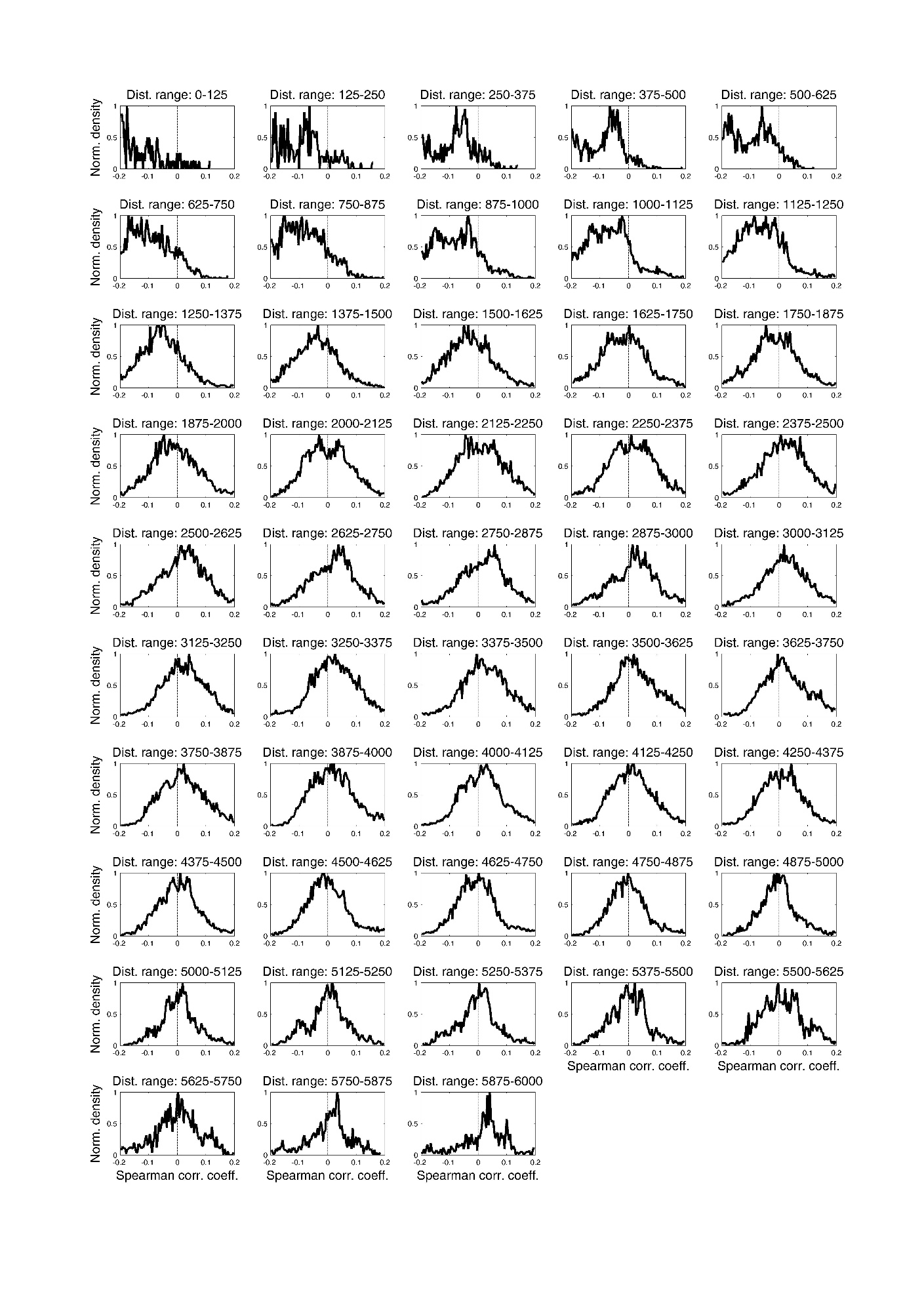


**Fig. S8a. Spearman correlation histograms.** Normalised histograms showing the Spearman correlation values in the x-axis and the normalized number of correlation estimations in the y-axis for distances ranging from 0 to 6000 km, with interval of 125 km, obtained for the band 2.5-5.0 s.


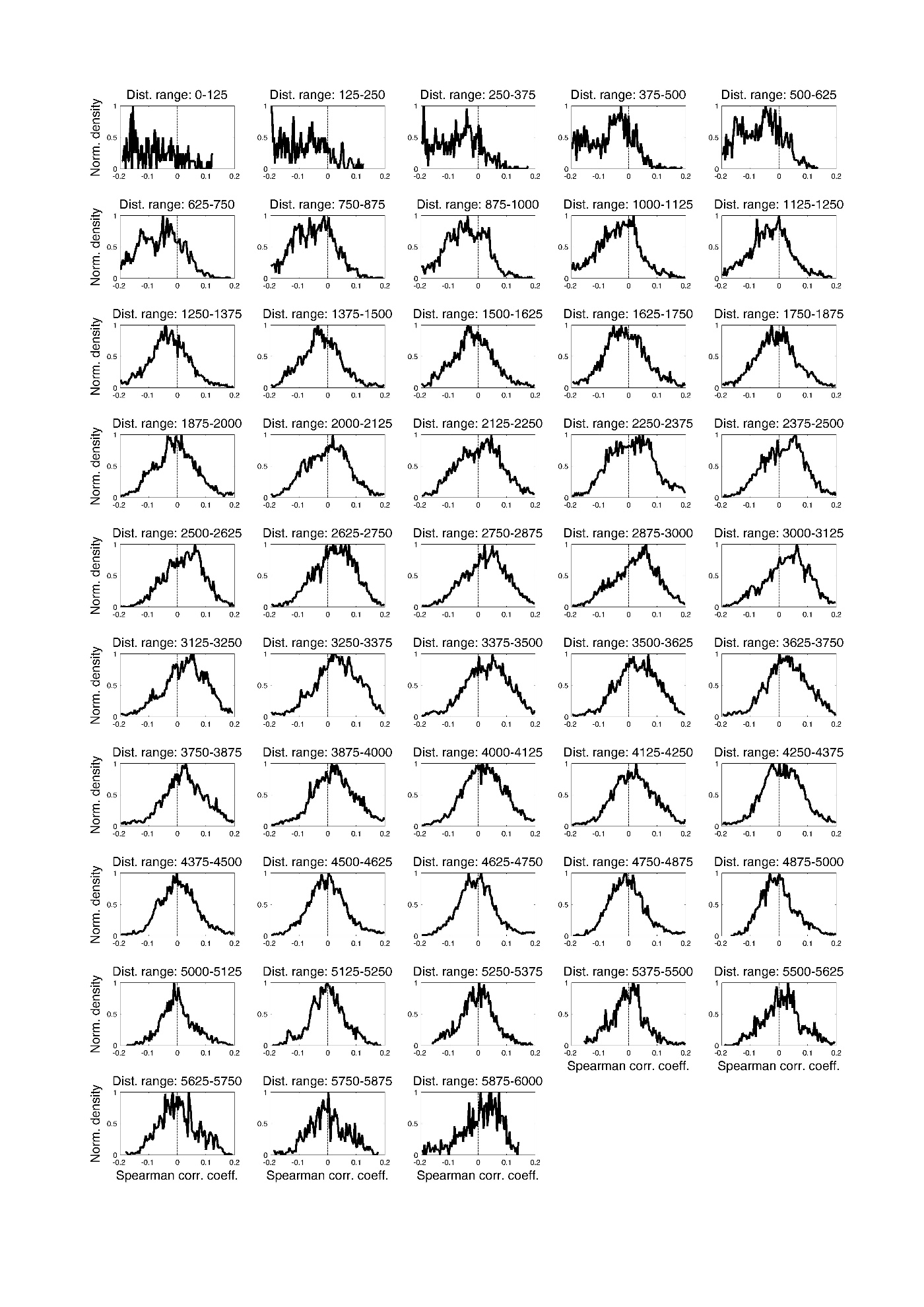


**Fig. S8b. Spearman correlation histograms.** Normalised histograms showing the Spearman correlation values in the x-axis and the normalized number of correlation estimations in the y-axis for distances ranging from 0 to 6000 km, with interval of 125 km, obtained for the band 5.0-10 s.


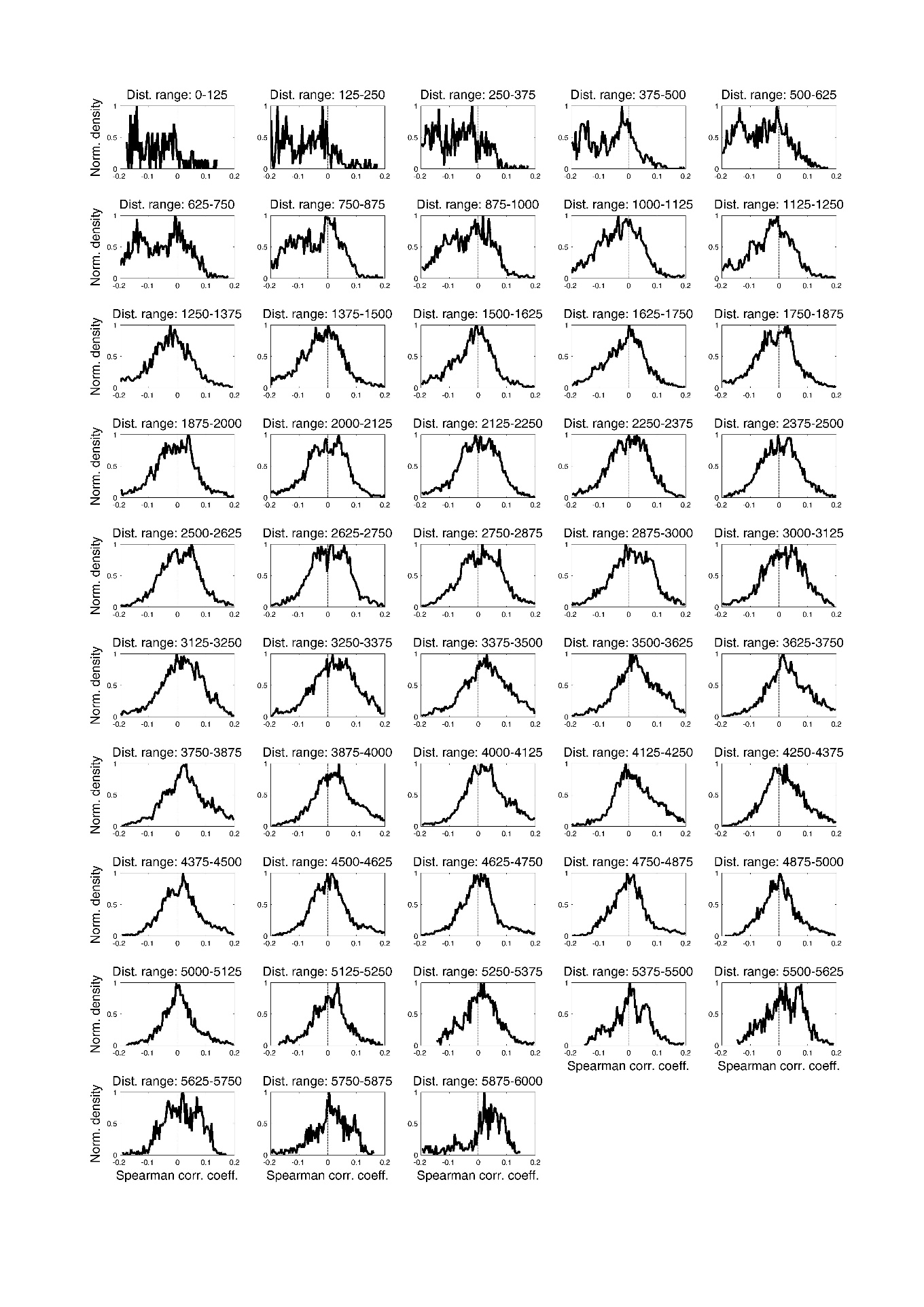


**Fig. S8c. Spearman correlation histograms.** Normalised histograms showing the Spearman correlation values in the x-axis and the normalized number of correlation estimations in the y-axis for distances ranging from 0 to 6000 km, with interval of 125 km, obtained for the band 13-20 s.


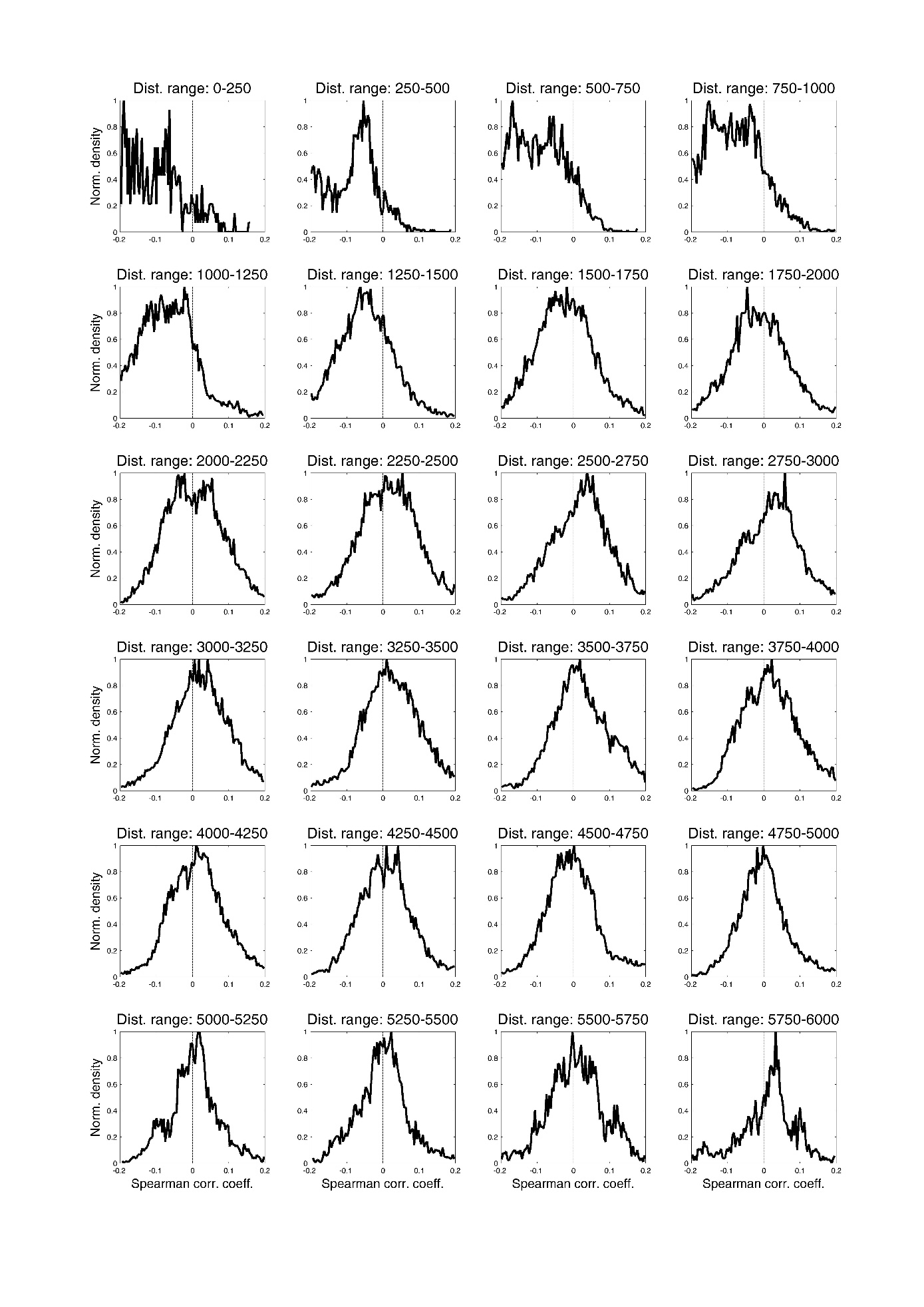


**Fig. S9a. Spearman correlation histograms.** Normalised histograms showing the Spearman correlation values in the x-axis and the normalized number of correlation estimations in the y-axis for distances ranging from 0 to 6000 km, with interval of 250 km, obtained for the band 2.5-5.0 s.


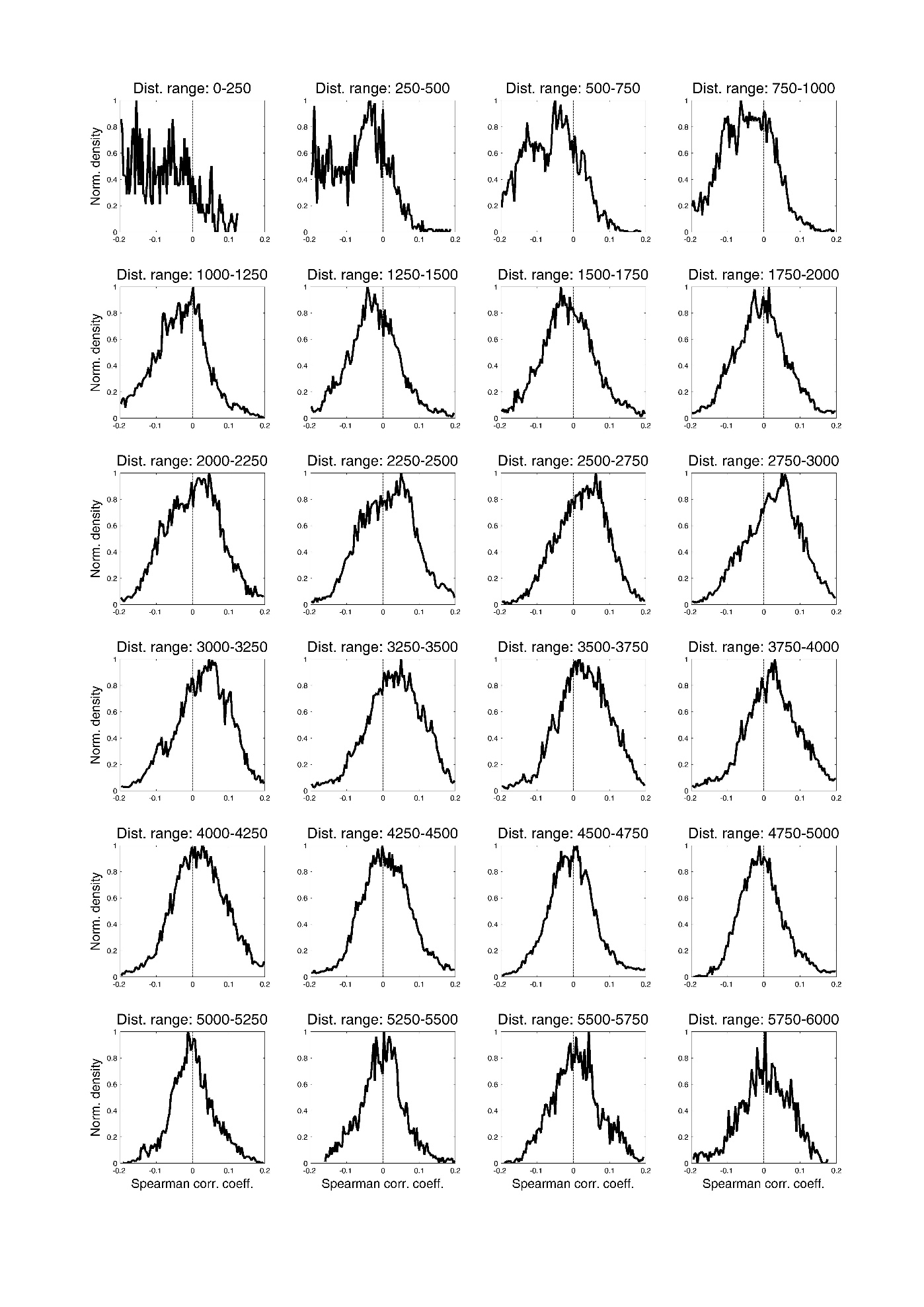


**Fig. S9b. Spearman correlation histograms.** Normalised histograms showing the Spearman correlation values in the x-axis and the normalized number of correlation estimations in the y-axis for distances ranging from 0 to 6000 km, with interval of 250 km, obtained for the band 5.0-10 s.

**
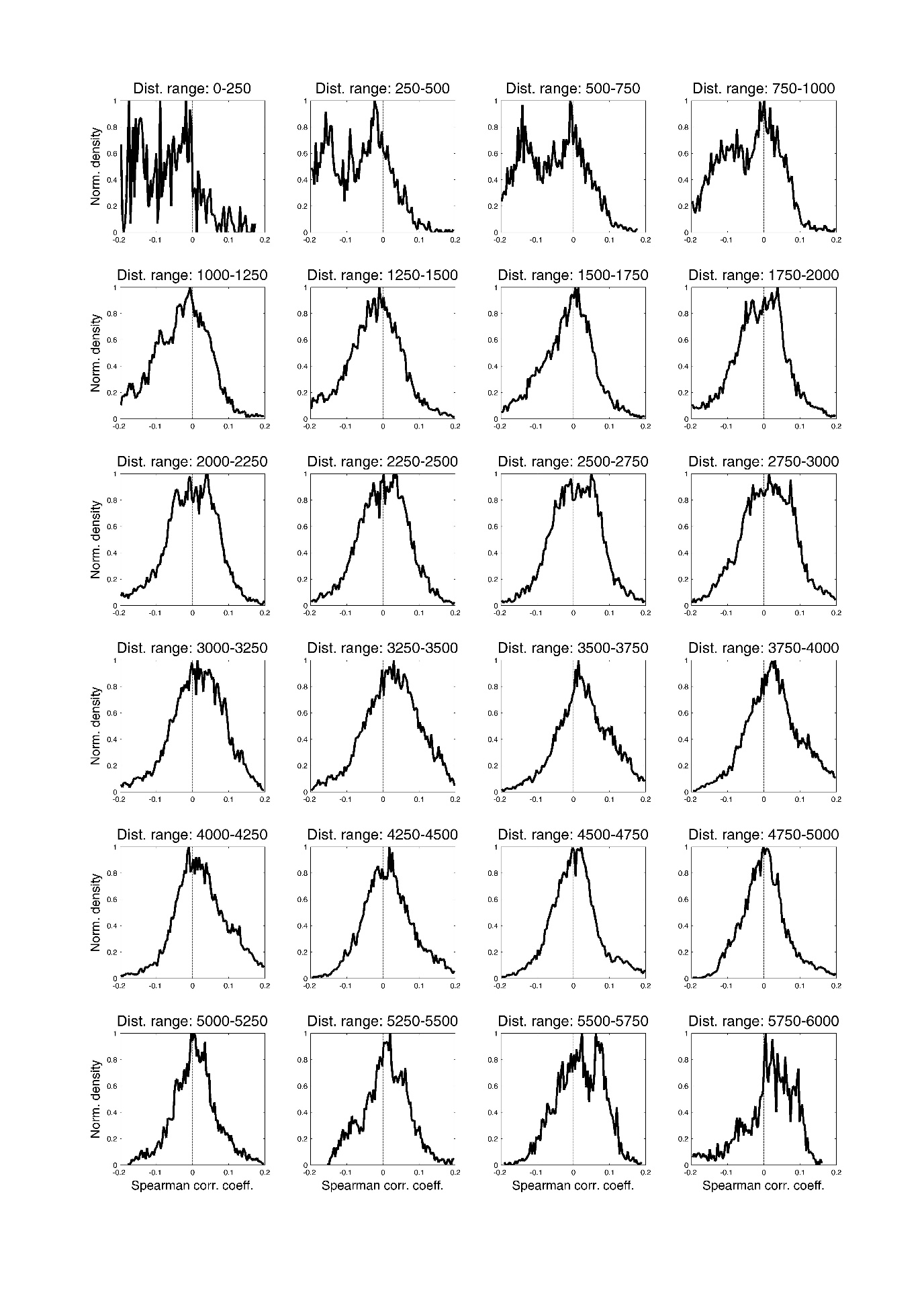
**

**Fig. S9c. Spearman correlation histograms.** Normalised histograms showing the Spearman correlation values in the x-axis and the normalized number of correlation estimations in the y-axis for distances ranging from 0 to 6000 km, with interval of 250 km, obtained for the band 13-20 s.


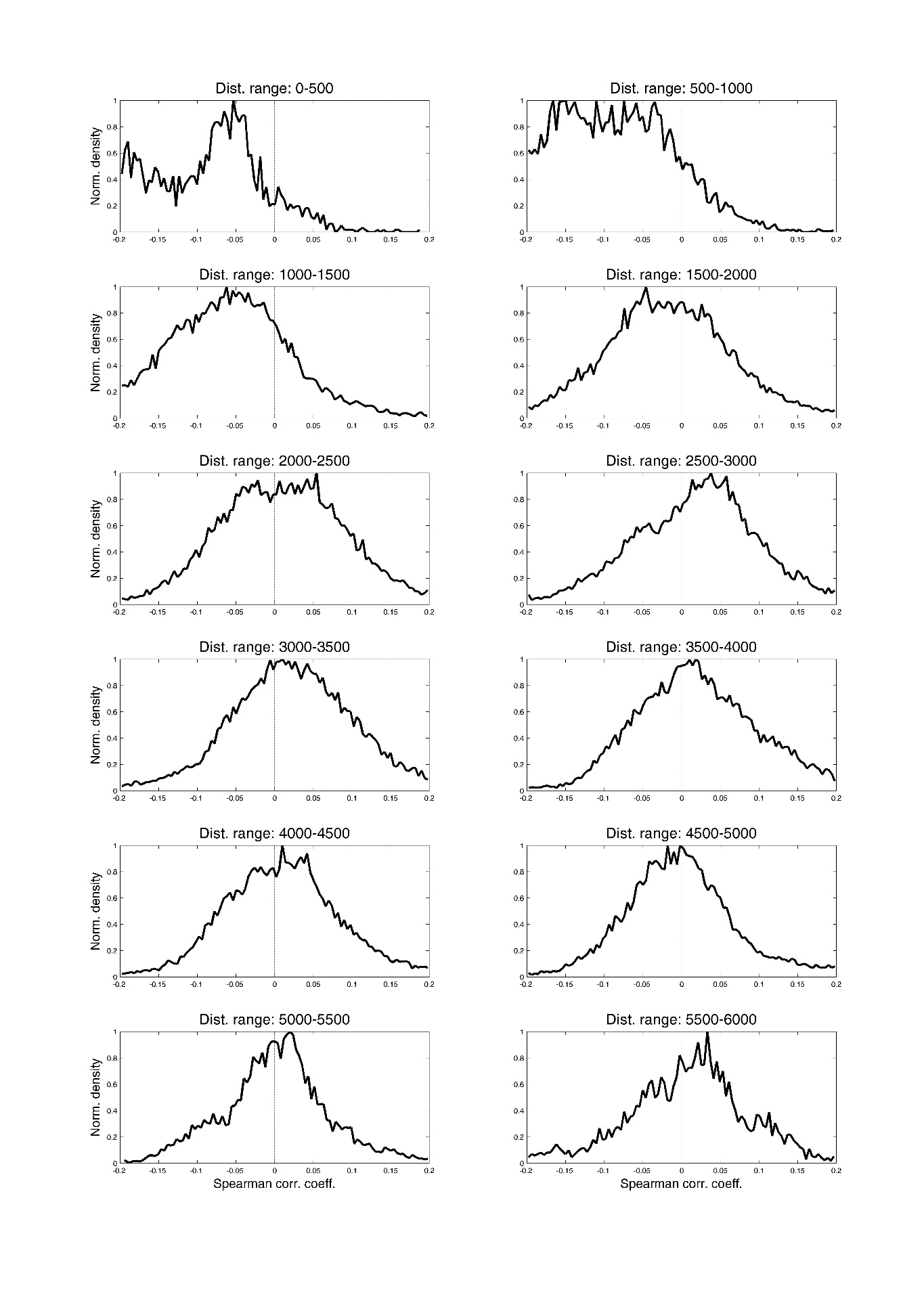


**Fig. S10a.** **Spearman correlation histograms.** Normalised histograms showing the Spearman correlation values in the x-axis and the normalized number of correlation estimations in the y-axis for distances ranging from 0 to 6000 km, with interval of 500 km, obtained for the band 2.5-5.0 s.


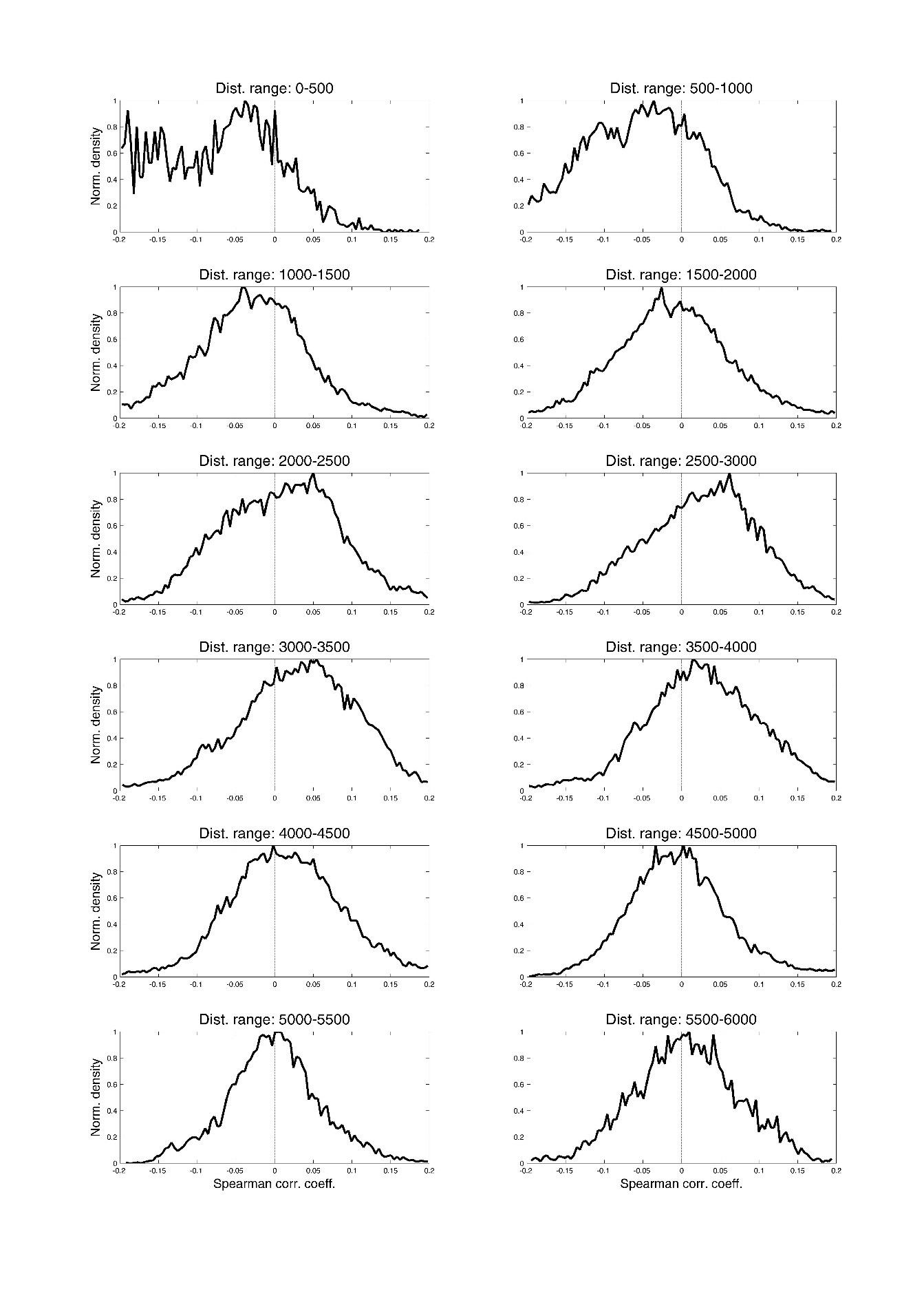


**Fig. S10b.** **Spearman correlation histograms.** Normalised histograms showing the Spearman correlation values in the x-axis and the normalized number of correlation estimations in the y-axis for distances ranging from 0 to 6000 km, with interval of 500 km, obtained for the band 5.0-10 s.


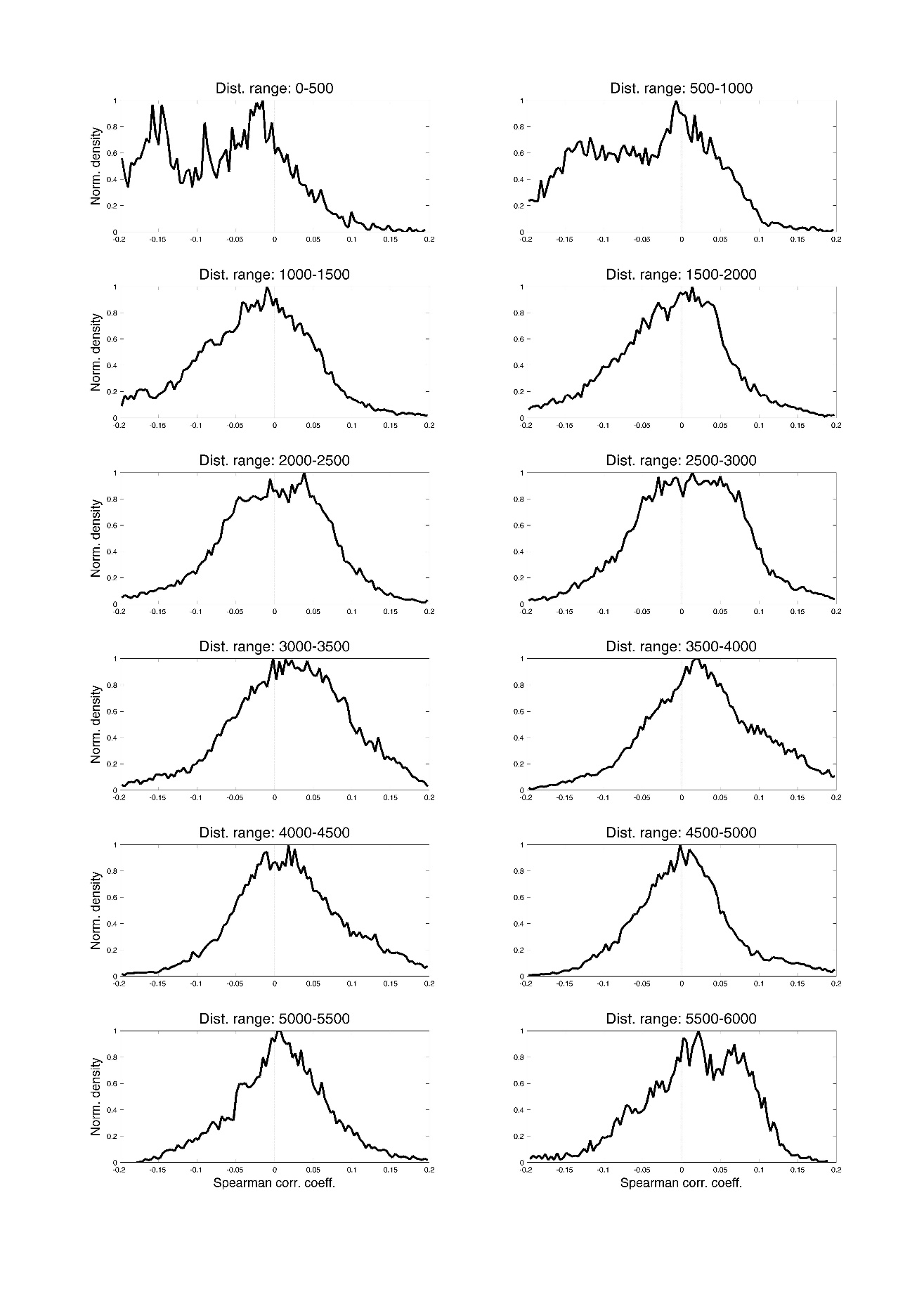


**Fig. S10c.** **Spearman correlation histograms.** Normalised histograms showing the Spearman correlation values in the x-axis and the normalized number of correlation estimations in the y-axis for distances ranging from 0 to 6000 km, with interval of 500 km, obtained for the band 13-20 s.


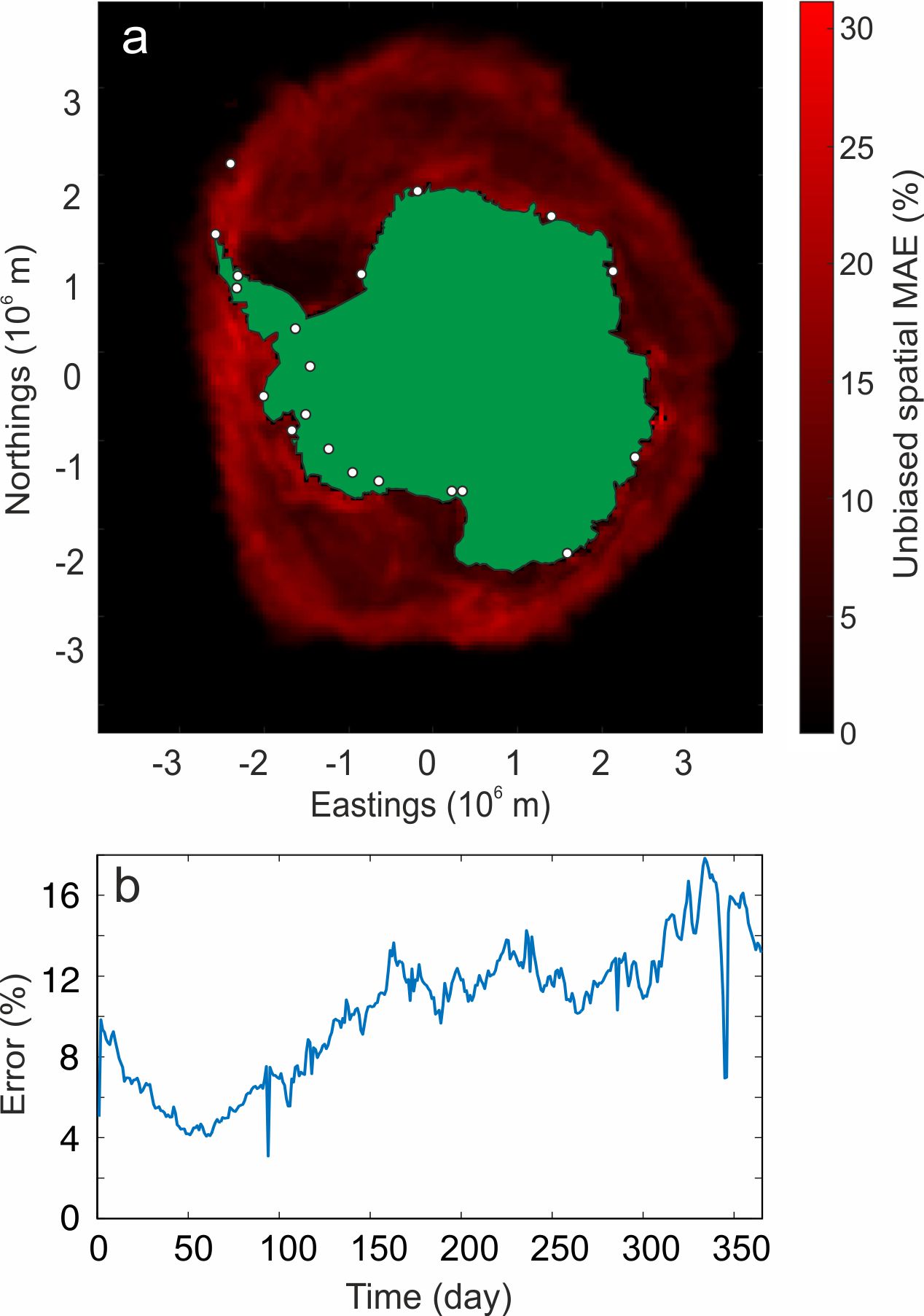


**Figure S11. Spatial and temporal distribution of error.** (a) Map showing the unbiased spatial mean absolute error (MAE) obtained through K-fold cross-validation. The white dots indicate the locations of the seismic stations. (b) Seasonal trend of the prediction error computed through K-fold cross-validation from the 1^st^ January to the 31^st^ December (see text for explanation about error computation). The map in (a) was created by Matplotlib package for Python (*61*).


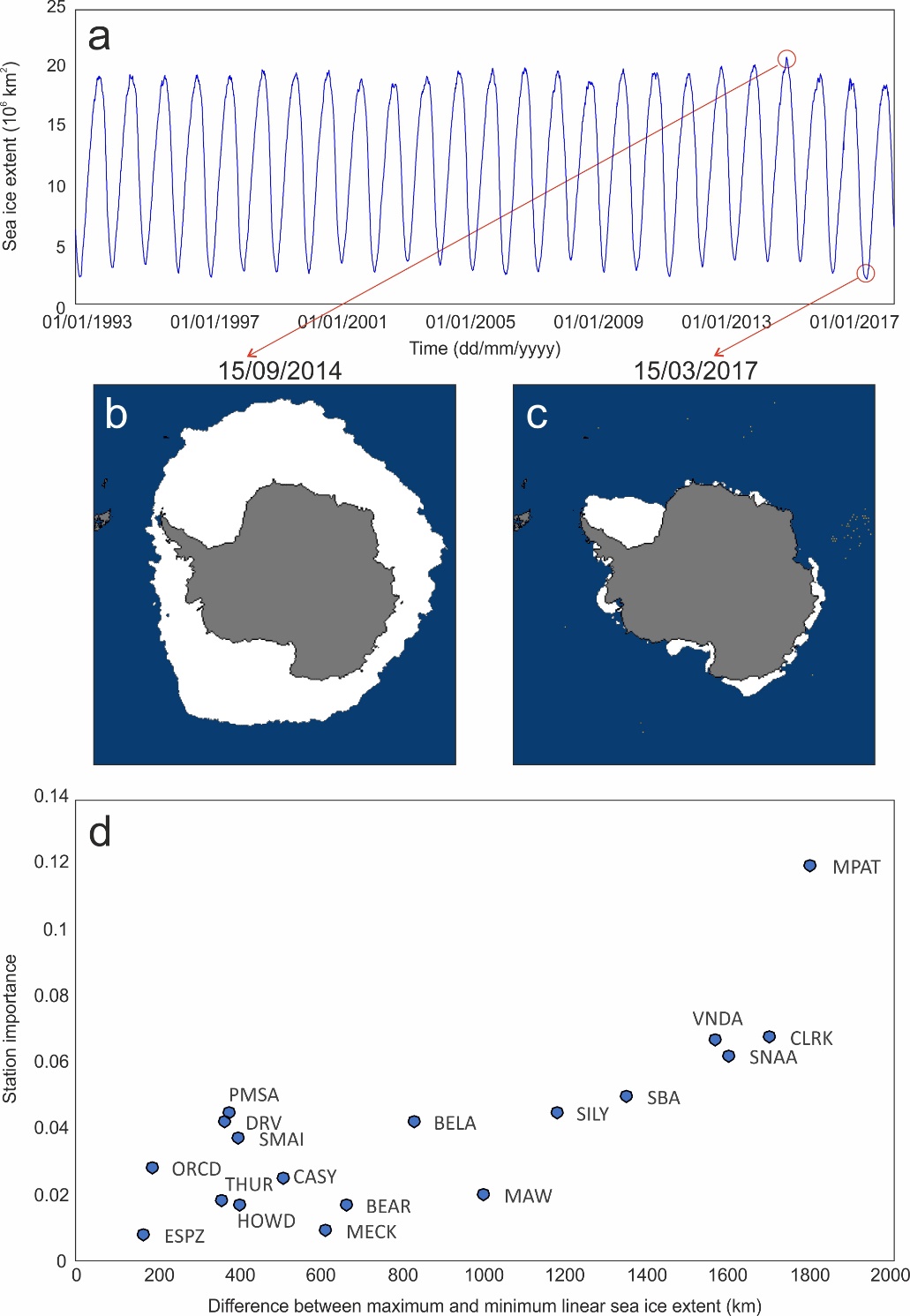


**Figure S12. Station importance versus time variation in sea ice extent.** (a) Temporal variation of sea ice extent during 1993-2017, computed by counting the daily number of grid cells with sea ice concentration higher than 15% and multiplying it by the cell area. (b,c) Maps of sea ice extent during 15 September 2014 and 15 March 2017, respectively. (d) Station importance, derived by the MLT, plotted versus the difference between maximum and minimum linear extent of the sea ice measured approximately normal to the coastline closest to the station during September 2014 and March 2017, respectively.

**Table S1. List of the** **MLT/IF** **performances.** List of the cross-validated performances of the different pairs MLT/IF sorted by ascending MAE plus its standard deviation ($\sigma_{MAE}$). “ExtraTrees” stands for Extremely Randomized Trees Regression, “RandomForest” for Random Forest Regression, “Knn” for K-Neighbors Regression, and “Linear” for Linear Regression. The “x” in the Time feature column means that the time-related feature was included in the training and cross-validation steps. The “-” in the Basic data means that only the time-related feature was included in the training and cross-validation steps (the microseism data were not used).
